# Visual aftereffects and sensory nonlinearities from a single statistical framework

**DOI:** 10.3389/fnhum.2015.00557

**Published:** 2015-10-13

**Authors:** Valero Laparra, Jesús Malo

**Affiliations:** Image Processing Lab, Universitat de ValènciaValència, Spain

**Keywords:** motion aftereffect, texture aftereffect, color aftereffect, adaptation, scene statistics, unsupervised learning, sequential principal curves analysis

## Abstract

When adapted to a particular scenery our senses may fool us: colors are misinterpreted, certain spatial patterns seem to fade out, and static objects appear to move in reverse. A mere empirical description of the mechanisms tuned to color, texture, and motion may tell us where these visual illusions come from. However, such empirical models of gain control do not explain *why* these mechanisms work in this apparently dysfunctional manner. Current normative explanations of aftereffects based on scene statistics derive gain changes by (1) invoking decorrelation and linear manifold matching/equalization, or (2) using nonlinear divisive normalization obtained from parametric scene models. These principled approaches have different drawbacks: the first is not compatible with the known saturation nonlinearities in the sensors and it cannot fully accomplish information maximization due to its linear nature. In the second, gain change is almost determined a priori by the assumed parametric image model linked to divisive normalization. In this study we show that both the response changes that lead to aftereffects and the nonlinear behavior can be simultaneously derived from a single statistical framework: the Sequential Principal Curves Analysis (SPCA). As opposed to mechanistic models, SPCA is not intended to describe *how* physiological sensors work, but it is focused on explaining *why* they behave as they do. Nonparametric SPCA has two key advantages as a normative model of adaptation: (i) it is better than linear techniques as it is a flexible equalization that can be tuned for more sensible criteria other than plain decorrelation (either full information maximization or error minimization); and (ii) it makes no a priori functional assumption regarding the nonlinearity, so the saturations emerge directly from the scene data and the goal (and not from the assumed function). It turns out that the optimal responses derived from these more sensible criteria and SPCA are consistent with dysfunctional behaviors such as aftereffects.

## 1. Introduction

Aftereffects are visual illusions which occur when the sensory system is put into a particular operation regime driven by the environment. After the adaptation to this environment, presented stimuli are perceived in unusual (unrealistic) ways.

**Figure 1:**
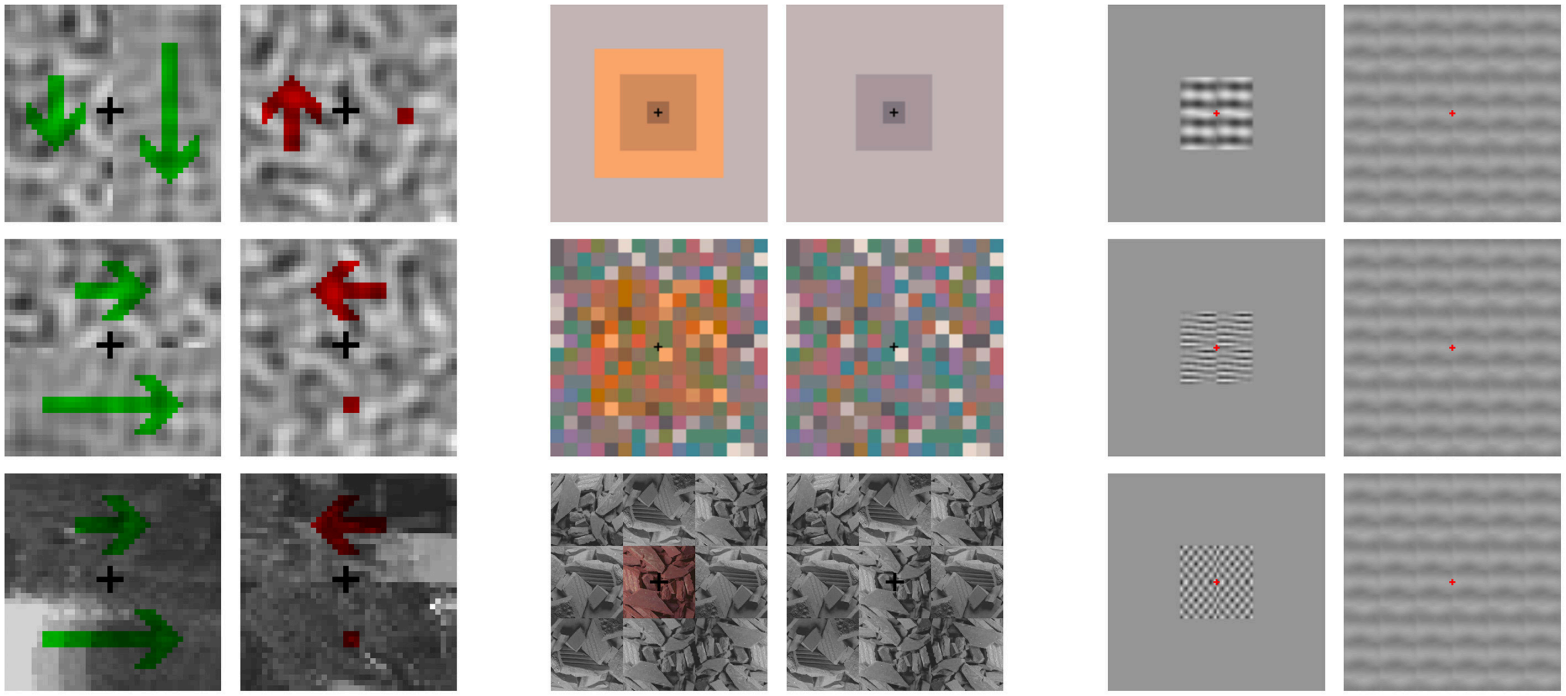
**Illustration of motion, color, and texture aftereffects**. The three panels show adapting stimuli on the left, and the test stimuli to be presented after adaptation are on the right. Visit http://isp.uv.es/after_effects/ for animated presentations of tests *after* the corresponding adaptors. The code to modify the parameters of these renderings is also available therein. **Left** (Motion) the first waterfall effect example (top) consists of a random noise sequence with different up-down speeds (represented by the green arrows) around the fixation point. When the sequence physically stops, the left part seems to move in the opposite direction while the right part is perceived as static (as represented by the red arrows). This effect works in any orientation (not only up-down) and also for natural images (second and third examples). **Center** (Color) color and contours of the scenes on the right of the panel change after adaptation to the scenes on the left: an illusory cyanish patch emerges around the fixation point in the physically stationary test due to the adaptation to the yellowish center of the previous stimulus. **Right** (Texture) while the contrast of the textures in the right column is stationary, it is perceived in different ways after adaptation to the high contrast achromatic textures on the left. Note that the strength of the induced blindness depends on the correspondence between frequency and orientation of the previous and post scenes.

Figure 1 illustrates classical motion, color and texture aftereffects. The static motion aftereffect (or waterfall effect) is the illusion of visual motion of a physically static pattern experienced after prolonged exposure to a moving pattern (Mather et al., 2008). After this adaptation, the static pattern seems to move in the opposite direction to the adapting stimulus. In the color aftereffect, exposure to an environment shifted toward a certain hue (either due to specific illumination, filters, or a biased distribution of reflectance), leads to the emergence of illusory colored contours (Loomis, 1972; Zaidi et al., 2012). In the example in Figure 1, cyanish regions appear around the fixation point. In the texture aftereffect, parts of stimuli with physically stationary contrast seem to fade out after prolonged exposure to localized high contrast patterns of similar frequency and orientation (Blakemore and Campbell, 1969; Barlow, 1990).

In the above cases, after exposure to certain context, decoding of new stimuli in a different context (interpretation of their representation) is incorrect. Previously proposed empirical models, such as the one reviewed below, describe the phenomenology through the appropriate response change, but do not address the fundamental question: why does the system behave in this dysfunctional manner?, or more specifically, what is the goal that leads to the kind of responses observed in the sensors and the way they change with the environment?

### 1.1. A classical description: gain control via divisive normalization

Spatio-temporal context (the presence of certain stimuli in the scene) leads to changes in the response of motion, texture, and color sensors (Ross and Speed, 1991; Foley and Chen, 1997; Hillis and Brainard, 2005; Morgan et al., 2006; Abrams et al., 2007). Contextual effects include adaptation and masking. Aftereffects arise when stimuli leading to a certain response in context 1, lead to a very different response in context 2. A sudden change in the environment would necessitate an immediate change of operation regime. Nevertheless, the required change in the sensors takes some time. Consequently, new stimuli are interpreted with the incorrect reference system leading to the illusory percept.

Adaptation and masking differ in the time and location of the stimulus driving the response change. However, these gain modifications are usually described in similar ways: for instance, divisive normalization has been used both for adaptation and masking with the appropriate parameter change (Ross and Speed, 1991; Foley and Chen, 1997). The currently accepted response models for motion, texture, and color perception (Watson and Solomon, 1997; Simoncelli and Heeger, 1998; Hillis and Brainard, 2005) include divisive normalization in a common *linear-nonlinear* structure: 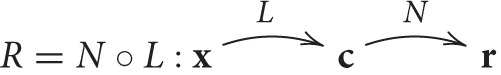 where *L* represents the application of linear receptive fields, $c_{i}=\underset{j}{\sum }L_{ij}x_{j}$, and *N* is the saturating divisive normalization gain control (Carandini and Heeger, 2012): a nonlinear transform in which the response of the *i*-th linear sensor, *c*_*i*_, is modulated by the response of the neighbor sensors of the linear stage in a certain spatio-temporal environment (simultaneous pedestal or previous adaptor), **c_a_**:

(2) $$r_{i}=N_{i}(c)=\frac{c_{i}^{\beta }}{1+\sum_{j}H_{ij}\;c_{aj}^{\beta }\;}$$

The key is that the response of a sensor is decreased by the current (or recent) activity of neighbor sensors, i.e., it is reduced by what is in its spatio-temporal context. In this introduction we only detail the classical description of the motion aftereffect based on the above model (Morgan et al., 2006) because similar arguments can be applied to the texture and color cases.

**Figure 2:**
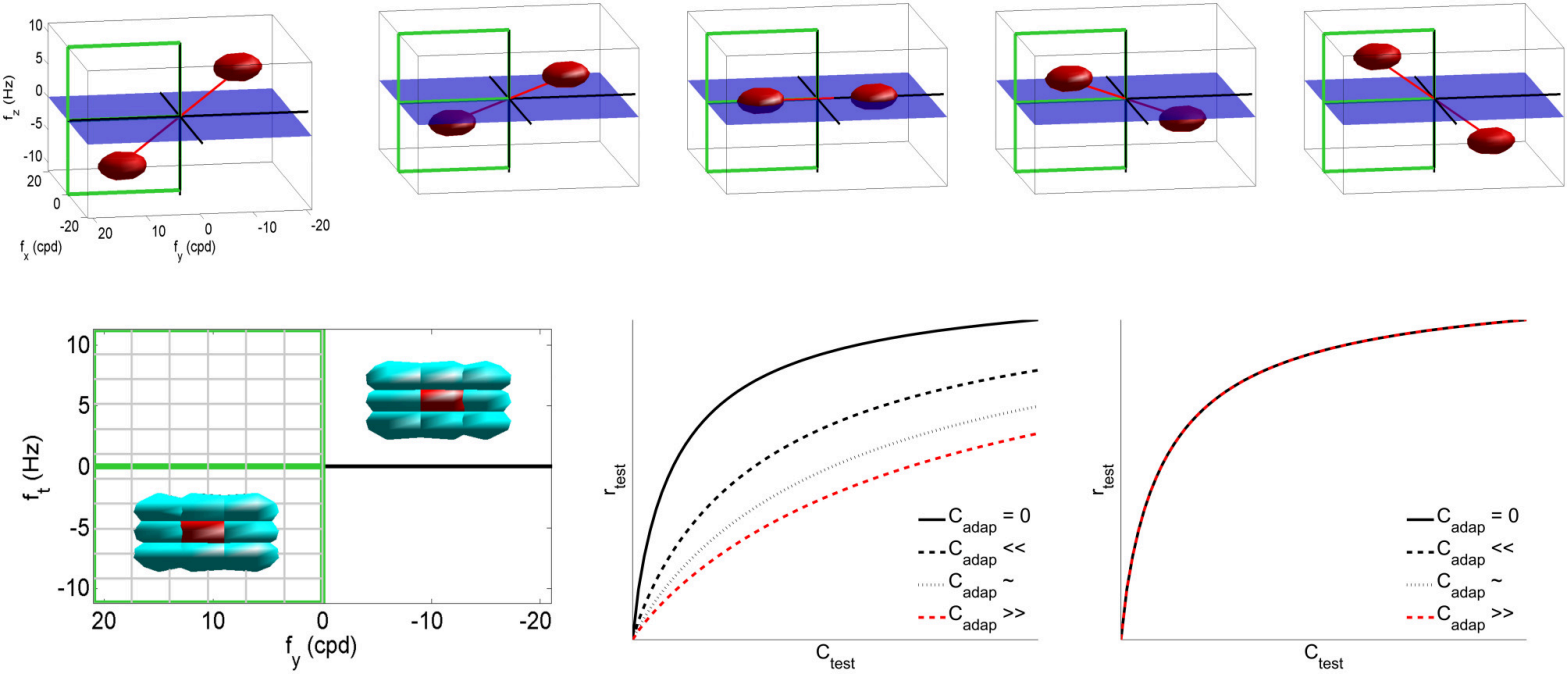
**The classical ***linear-nonlinear*** V1 model to describe the motion aftereffect**. Code for the empirical model and the experiment in this section is available at http://isp.uv.es/after_effects/. **Top** row shows examples of the spatio-temporal frequency response of sensors in the linear part of the motion sensing model, *L*. As stated in the text, such filters tile the visible region of the spatio-temporal Fourier domain. **Bottom left** plot shows the neighbor/interaction sensors (in cyan) of a particular sensor (in red), determined by the extent of *H*_*ij*_ in *N*. Given the symmetry of the Fourier domain, in the following we will focus on the response of the filters tuned to the frequencies shown in the semi-plane highlighted in green (positive and negative vertical speeds). The nonlinear plots display the response of the *i*-th nonlinear sensor as a function of the amplitude of the *i*-th linear response, for progressively higher neighbor activities *C*_*j*_ in the case of a similar test and adaptor (center) or a very different test and adaptor (right).

In the case of motion vision (Heeger, 1987; Simoncelli and Heeger, 1998), **x** is a vector with the irradiance at each pixel of a video patch, and *L* contains the receptive fields of local spatio-temporal frequency analyzers. Then, the response of these filters is normalized by a linear combination of the neighbor responses, Equation (2) (Simoncelli and Heeger, 1998). In this numerical example we used video patches of 32 frames of spatial size 16 × 16, with spatial and temporal sampling frequencies of 48 cycles/deg and 24 Hz, respectively. We built a set of V1-like receptive fields using spatio-temporal Gabor band-pass filters (Watson and Ahumada, 1985; Heeger, 1987) tuned to a uniform cartesian grid of 11 × 11 × 11 spatio-temporal frequencies in the range *f_x_* = [−18, 18] cpd and *f*_*t*_ = [−10, 10] Hz, with bandwidths of 2 cpd, and 1.5 Hz (see examples at the first row of Figure 2). Moreover, the neighborhood *H* was restricted to the eight closest filters in the frequency domain (bottom-left plot in Figure 2), and all the responses in the neighborhood contributed to the nonlinear attenuation in the same way. The excitation-inhibition exponent was set to β = 0.9 to ensure saturation even with no activity in neighbor sensors. As a result, the response of the nonlinear mechanisms reduces with the activity of the neighbors (bottom-center plot in Figure 2). However, the response stays the same if the context is far from the tuning band of the sensor and its closest neighbors (bottom-right plot). Similar behavior is obtained for other values of neighborhood and exponent. Particular values are irrelevant; only relevant is the fact that this attenuation (intrinsic to divisive normalization) will give rise to the aftereffect, as shown below.

**Figure 3:**
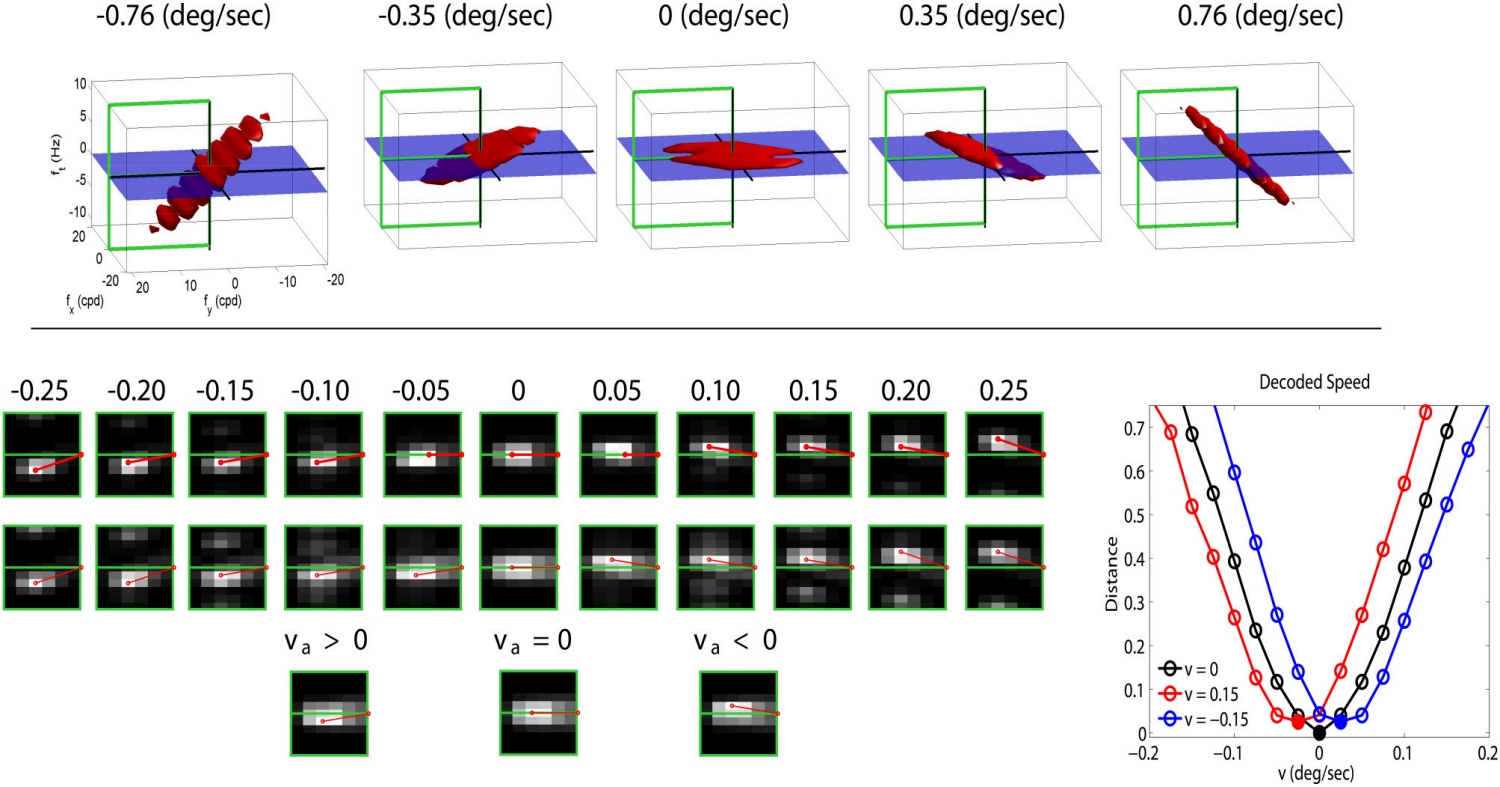
**Motion aftereffect in sensors with divisive normalization**. **Top** (stimuli) shows examples of noise sequences moving at different vertical speeds in the Fourier domain. Due to the properties of 3-*d* Fourier transform, speed induces progressive inclination in the spectrum with regard to the *f*_*t*_ = 0 plane. We used a cut-off frequency of 18 cpd to prevent aliasing. **Bottom-left** (responses) *first row* shows the linear responses of the sensors tuned to frequencies in the (0, *f*_*y*_, *f*_*t*_) plane for a series of noise sequences (such as those in the top panel) with the speed represented by the numbers (in deg/sec). The response of the filters is scaled by the standard deviation of the amplitude of natural sequences. This is just a convenient *contrast* definition so that response amplitudes roughly cover the [−1, 1] range for every frequency. Consistently with the spectra, speed induces progressive imbalance in the linear responses above and below the *f*_*t*_ = 0 plane. *Second row* shows the corresponding nonlinear responses when no prior adaptation is considered, i.e., *c*_*a*_ = *c* in the denominator of Equation (2). The nonlinear stage also presents a characteristic correspondence between imbalance of the response pattern and speed. *Third row* shows the response pattern to a static input in three different adaptation situations: adaptation to positive speed (**v_a_** > 0), no prior adaptation (**v_a_** = 0), and adaptation to negative speed (**v_a_** < 0). **Bottom-right** (speed decoding plot) shows the distances (Equation 3) between the responses to static patterns (3rd row in the response panel) and the set of possible responses under no adaptation (2nd row in the response panel). A Different line style corresponds to a different adaptation state (zero, positive, and negative) and the minimum indicates the decoded speed (zero, negative, and positive). The sign reversal is the motion aftereffect.

The top panel of Figure 3 shows a range of stimuli in the Fourier domain with different speeds (in deg/sec). The bottom-left panel with gray-level plots (lighter gray means higher activation) displays the corresponding linear and nonlinear responses of the model (in the highlighted semi-plane) when assuming no prior adaptation (first and second rows of the response panel). In this neutral operation regime (no prior adaptation) static patterns lead to balanced responses above and below the *f*_*t*_ = 0 plane. For greater positive and negative speeds there is a progressive imbalance in the response pattern toward sensors above or below the *f*_*t*_ = 0 plane. This specific imbalance is the feature associated to a certain speed. According to Heeger (1987), given a test sequence, the speed is estimated by minimizing the distance between the (actual) responses elicited by the sequence, *r*_*i*_, and the (theoretical) responses associated with white noise patterns of different uniform speeds, $r_{i}^{theor}\left(v\right)$ in a neutral adaptation condition:

(3) $$\hat{v}=\underset{v}{\min}\sum_{i}{\left(r_{i}-r_{i}^{theor}(v)\right)}^{2}$$

This straightforward decoding strategy to infer the speed from the responses, together with the inhibitory properties of the nonlinear responses, gives rise to the waterfall aftereffect. This can be seen by considering the responses elicited by a static stimulus in (1) the neutral adaptation state (second row in the gray-level response panel in Figure 3), and (2) when adapted to positive or negative speeds, ±0.15 °/s, respectively, (third row in the response panel in Figure 3). The decoded speed comes from the comparison of the actual responses with the (theoretical, neutral adaptation) patterns in the second row.

In the neutral adaptation state, a static stimulus leads to a balanced set of responses (center). Therefore, distance with regard to the theoretical patterns, Equation (3) (black line in the decoding plot at the bottom-right of Figure 3), is minimum for **v** = 0, so the stimulus is (correctly) interpreted as static.

However, if this sensory system is exposed to **v** = ±0.15 °/s, the transform *N* is driven by imbalanced linear responses **c_a_** (similar to those in the 3rd and 9th positions in the 1st row of the response panel). In that situation, according to the behavior shown in Figure 2, the responses of some sensors will be reduced: in our case, those slightly above (or below) the *f*_*t*_ = 0 plane. As a result, when the static stimulus is presented after adaptation to a moving-up (or moving-down) pattern, the nonlinear response is the one presented on the left (or right) of the third row in the Figure 3 response panel. When comparing these responses (those of the third row) to the theoretical responses above (second row), one gets the *shifted* differences in red and blue in the decoding plot. Therefore, the standard decoding procedure after adaptation implies that static patterns are (incorrectly) perceived as moving down or up, respectively.

In summary, the trends of the static motion aftereffect are robustly obtained from the divisive normalization description. The shift of the decoding distance curves in Figure 3 is linked to the attenuation in Figure 2, which is intrinsically associated to divisive normalization. Note that we did no specific parameter optimization to achieve the illusion: we only selected the simplest possible neighborhood, and a reasonable exponent. This means that assuming divisive normalization (almost automatically) leads to the static motion aftereffect. It is true that in order to reproduce fine details of the effect, two cascaded adaptation mechanisms may be required (Stocker and Simoncelli, 2009): (1) one acting on the neighbors of zero speed sensors, and (2) the other affecting the neighbors of the sensor tuned to the adaptor speed. The second is the one considered in our example. However, it is important to note that both mechanisms are isomorphic and can be implemented though divisive normalization. And more importantly for this introductory illustration, according to the results in Stocker and Simoncelli (2009), the most relevant mechanism to describe the aftereffect is the second one.

The speed decoding scheme in our example, Equation (3) following (Heeger, 1987), is convenient because of its simplicity, but it is not realistic since it involves comparisons with theoretical response patterns that have been learned and stored *somehow*. However, note that the key issue is the change in the operation regime in the encoder, leading to a mismatch with the decoder, and not the particular decoding scheme. In this work we focus on the changes in the encoder and the reasons behind them (organization principles). We do not address the specific decoding mechanism: possibilities include the qualitative reasonings in Morgan et al. (2006), or the more quantitative approaches in Series et al. (2009), but in both cases, the outputs of the adapted encoder would be misinterpreted by the (temporarily unaware) decoder.

The empirical model in Equations 1, 2 also applies to texture and color perception, and also describes the considered aftereffects. In the case of texture vision (Watson and Solomon, 1997), **x** is a vector with the irradiance at each pixel of an image patch, and the matrix *L* contains the receptive fields of local spatial frequency analyzers. According to this, *c* is a local-frequency transform of **x** (e.g., local DCT, Gabor transform…). Then, the sigmoidal transform, *N*, modifies the output of the frequency analyzers depending on the activity of the neighbors in the immediate past. The perceived texture depends on the relative amplitude of these responses. As a result, attenuation of certain responses implies a reduction in the perceived contrast of the corresponding patterns. Similarly to the motion-tuned filters used above (Figure 2), in texture sensors interaction is greater between close frequencies/orientations and spatial locations (Watson and Solomon, 1997; Laparra et al., 2010; Malo and Laparra, 2010). Therefore, after prolonged exposure to a certain frequency and orientation in a particular spatial location, sensors tuned to those features are put in a low response regime. This explains the induced texture fade-out illusion.

In the case of color vision (Fairchild, 2005; Hillis and Brainard, 2005), **x** contains the values of the spectral radiance coming from a small region of the scene, the matrix *L* involves the color matching functions of sensors tuned to short, medium and long wavelengths, and an opponent channel transform. Standard color appearance models include Von Kries-like divisive normalization (scaling by the *white* -or average color in a neighborhood-) prior to the computation of opponent channels (Fairchild, 2005). Therefore, **c** are 3-*d* vectors with an achromatic response and two chromatic responses, red-green and blue-yellow. The origin of the chromatic plane depends on the adaptation signal used in the Von-Kries normalization. In the next stage, the sigmoidal transform, *N*, modifies each response according to its amplitude with regard to the origin (or adaptation point). Perceptual descriptors of color are obtained from the nonlinear response, **r**. For instance, hue depends on the signs and the proportion of the nonlinear response of the chromatic channels. Changing the (white) adaptation point in the empirical models implies shifting the nonlinear responses (Krauskopf and Gegenfurtner, 1992; Laparra et al., 2012). Therefore, after prolonged exposure to an environment with white bias, a stimulus with flat spectral radiance (which used to be at the origin in neutral adaptation conditions) is perceived as having a certain hue and non-zero saturation (non-zero response in the opponent channels due to the shift in the curves).

The above examples show that aftereffects are a by product of shifts in response. In addition to the aforementioned divisive mechanisms, subtractive mechanisms have also been proposed to describe the sensitivity loss (Adelson, 1982; Ferwerda et al., 1996; Morgan et al., 2006). Beyond the specific parametrization, Shapley and Enroth-Cugell explicitly pose the *why* question: in their review on light adaptation (Shapley and Enroth-Cugell, 1984) they list some functional advantages of adaptation (purposes, or reasons why it is desirable). In particular, they mention (i) keeping the sensitivity constant for high dynamic range inputs, and (ii) making the contrast computation illumination invariant. These advantages are also discussed in Dahari and Spitzer (1996), and color constancy is another related advantage pointed out in Spitzer and Semo (2002). The models following (Dahari and Spitzer, 1996), using nontrivial semisaturation constants in divisive normalization, not only have these functional advantages, but also reproduce other visual illusions such as chromatic induction (Spitzer and Barkan, 2005) and chromatic Mach bands (Tsofe et al., 2009). Nevertheless, note that the simple statement of the purpose of a behavior (while giving the appropriate qualitative insight) is not a principle from which to derive the behavior. In this sense, functional advantages typically discussed in the literature are true, but they are not actual explanations of the behavior. This is the classical difference between descriptive/mechanistic models that address the *what*/*how* questions, and normative models focused on the *why* question through derivations from first principles (Dayan and Abbott, 2005).

Here we follow a normative tradition (Barlow, 1990; Wainwright, 1999; Weiss et al., 2002; Stocker and Simoncelli, 2006; Coen-Cagli et al., 2009), and we obtain optimal sensors according to infomax/errormin criteria by computing non-Euclidean Principal Curves on data from natural scenes. Since we find that these optimal sensors have the behavior that leads to the aftereffects, these illusions are not arbitrary failures of the system, but they can be explained as the result of optimal operation regimes.

### 1.2. Aftereffects and statistical shifts of the reference system

Qualitative interpretation of the response induced by a test depends on its location in a feature space, but locations depend on *where* and *what* the reference system is. The key to decoding the meaning of the responses is the (vector-like) basis of the feature space. Empirical models *describe* how changes in the environment induce changes in the sensors that constitute the reference axes of the representation space. If reference axes change, stimuli are perceived in different (sometimes unrealistic) ways. Nevertheless, empirical models *do not explain why* reference axes change in this way.

Changes in interpretation due to changes in the reference system is an old idea in psychology (Helson, 1948). Nevertheless, these classical theories lack a quantitative reason *why* these changes occur. According to his *Efficient Coding Hypothesis*, Horace Barlow suggested that visual aftereffects may come from a reference axes adaptation policy that tries to minimize correlation between responses thus maximizing the use of the dynamic range of the sensors (Barlow, 1990). Even though he suggested a quantitative explanation (the link between information theoretic principles and aftereffects), he did not provide explicit algorithms to implement this explanation nor the statistical data to train the theoretical sensory system. The first complete interpretation of statistics-driven adaptation was *linear* and *second order* (Wainwright, 1999): the linear gain that *maximizes the transmitted information* depends on the average spectra of signal and noise. Even though Wainwright uses linear (restricted) techniques, he suggests that illusions are a reflection of changes in the optimal response after the changes in the signal. Here we follow the same general idea but using a nonlinear manifold equalization method that does not suffer from the (too strong) linear and second order constraints associated with decorrelation. Following the success of statistical explanations of neural organization in fixed environments using higher order independence (Simoncelli and Olshausen, 2001; Hyvarinen et al., 2009), current research on adaptation to dynamic environments (Clifford et al., 2007; Schwartz et al., 2007; Mather et al., 2008) stresses the need for tools that learn according to well-defined statistical principles.

Examples of statistical links between changes in the signal and the perceptual reference system include spatio-chromatic receptive fields (Gutmann et al., 2014), and purely chromatic sensors (Laparra et al., 2012) in different illuminations. In the same way, unsupervised learning with well-defined goals should be used to explain aftereffects in scenes with unusual motion, contrast, illumination, or reflectance distribution. Specifically, understanding aftereffects requires multidimensional equalization, as suggested, but not explicitly implemented in Clifford et al. (2000) and Clifford (2002). This is the normative approach we propose in this work: we show that an appropriate unsupervised learning tool, the Sequential Principal Curves Analysis (SPCA) (Laparra et al., 2012), captures the statistical trends in natural movies, textures, and colors, which explain the aftereffects. As opposed to previous normative approaches (Barlow, 1990; Webster and Mollon, 1997; Wainwright, 1999; Gutmann et al., 2014), that use linear techniques to maximize independence and match the manifolds in different environments, SPCA is a more flexible, not necessarily linear, equalization. As a result, it can also account for the experimental saturation found in the motion, texture, and color sensors. As opposed to Abrams et al. (2007), Coen-Cagli et al. (2009), Schwartz et al. (2009), and Series et al. (2009), who can account for the saturation because they already start from parametric models that include the appropriate nonlinear expression, SPCA is nonparametric, so it does not assume any particular response shape. Therefore, contextual saturations come from the scene data and the statistical goal, but not from the assumed parametric model.

The paper is organized as follows: Section 2 analyzes the structure of the linear responses to natural signals (motion, texture, color) after stage *L* in Equation (1). The particular signal statistics together with the organization strategies in SPCA give rise to nonlinear sensors with different operation regimes depending on the environment, thus leading to the aftereffects. In Section 3 we discuss related research and stress the advantages of using flexible nonparametric learning to better state that sensible organization principles lead to aftereffects. Finally, the methodological Section 4 reviews the technical differences with conventional decorrelation and explicitly checks the ability of SPCA to obtain either nonlinear independent components or noise-robust representations for spatial textures. The accuracy of the proposed transform (and inverse) and its success in optimizing the considered design strategies confirms the validity of the results for the aftereffects.

## 2. Results

In the *linear* + *nonlinear* context of Equation (1), the considered illusions come from environment-driven changes of the response after the first linear stage. There is an extensive literature that derives the linear receptive fields from the signal statistics, so we can build up from there. Therefore, in each experiment we start from a certain first linear stage (motion, texture, or color), and then we derive the nonlinear behavior of the corresponding second stage from scene statistics. The linear stage (the application of a set of linear analyzers) can be seen as a change of representation. The responses of the linear analyzers describe the input samples in a new (transformed) representation. We will use these transformed samples as the statistical set to train the second stage. These are the linear analyzers that we use in each experiment:

- In the motion vision case, linear Principal Component Analysis (PCA) and linear Independent Component Analysis (ICA) applied to natural movies lead to narrow band-pass filters in the spatio-temporal Fourier domain (Hyvarinen et al., 2009). Therefore, the spatio-temporal Gabor filters used in the dynamic V1 models, such as the one used in Section 1.1 (Heeger, 1987; Simoncelli and Heeger, 1998), can be understood by efficient coding arguments (van Hateren and Ruderman, 1998; Hurri and Hyvärinen, 2003). In the motion experiments below we assume that the linear stage consists of the Gabor filters used in the illustration in Section 1.1. Then, we will consider the statistics of the joint responses of selected filters to natural sequences. These linear responses to natural movies constitute the training set for optimal SPCA sensors.
- In the spatial texture case, simple decorrelation of natural luminance patterns leads to frequency selective filters (Hancock et al., 1992). Even though linear ICA leads to more accurate descriptions of static V1 receptive fields (Olshausen and Field, 1996; Hyvarinen et al., 2009), when dealing with small image patches linear PCA is a convenient approximation for the first stage (Malo et al., 2006). In the texture experiments below we will consider the statistics of the responses of linear PCA filters and we will train the nonlinear SPCA using natural images transformed using PCA. Note that this convenient choice is sensible not only because the resemblance of local PCA filters to V1 frequency analyzers, but also because PCA is a simple rotation that does not distort the data structure in any way.
- In the color vision case, simple decorrelation of tristimulus values also gives rise to perceptual-like opponent channels (Buchsbaum and Gottschalk, 1983). The consideration of spatial information and higher order relations does not change the emergence of spectrally opponent sensors (Gutmann et al., 2014). In the color experiments below we assume the linear *achromatic, red-green*, and *yellow-blue* mechanisms in Ingling and Tsou (1977) not only because they reproduce basic color psychophysics (Capilla et al., 1998) but also because these sensors can be easily understood using efficient coding (PCA). We consider the statistics of the responses of the linear Ingling and Tsou sensors in different environments. These are the samples for the SPCA training.

In every case (motion, texture, color) the results consist of two parts: (1) explicit display and analysis of the scene data after the linear transforms, and (2) derivation of the different behaviors in the nonlinear responses that induce the aftereffects.

Explicit display of the data after the linear transforms, which is not usual in the aftereffect literature, stresses the similarities of the three modalities analyzed here. In all three modalities the observed data distributions are similar. Therefore, if the goal of the nonlinear stage is related to equalization, the stage will present similar saturating responses in the three modalities. Here, equalization means that highly populated regions in the input space are expanded, while the low density regions are shrunk, in the response domain. Independently of the technique used to implement the equalization, the intuition obtained from the data has value in itself since it justifies the similarities of the psychophysics of the three modalities (Clifford et al., 2000; Clifford, 2002).

Here we employ SPCA as the nonlinear step as it can be tuned for different forms of multidimensional equalization (depending on the statistical goal): *information maximization* and *error minimization* (Laparra et al., 2012). The computation of the nonlinear responses in SPCA is analogous to the computation of the responses of a set of linear analyzers. In the linear case, the response of each sensor to a stimulus is just the projection of the sample onto a straight line defined by the receptive field. In the nonlinear case we substitute the straight lines with curves, so the response of each sensor is just the projection onto the corresponding curve. In the linear approaches PCA or ICA are used to compute the straight lines (linear sensors), and here we compute the curves (nonlinear sensors, or curvilinear reference axes) by drawing a set of Principal Curves. Section 4 and the online Supplemental Material describe how this set of nonlinear sensors and the corresponding projections are computed, and how to control the design criterion. In every modality, we compute the nonlinear responses using different goals for SPCA and scene data from different environments. In the described setting, changes of environment are characterized as different degrees of activation of neighbor linear sensors. Different activation may come from biased motion, high exposure to certain texture patterns, or changes in the chromatic properties of the scene (reflectance/transmittance or illumination). SPCA follows statistically meaningful principles to show that different environments induce different operation regimes or different reference axes. As stated in the introduction, these differences in operation regime lead to the aftereffects. Motion, color, and texture calibrated stimuli (training data), general code for SPCA, and specific code to reproduce all the results are available online: http://isp.uv.es/after_effects/.

### 2.1. Motion aftereffect from SPCA

#### 2.1.1. Natural video data after the linear stage

In order to study the statistics of visual motion we considered samples from raw (undistorted) natural sequences. We used the achromatic channel of 25 undistorted video clips from the publicly available VQEG and LIVE databases (Webster and Brunnstrom, 2000; Seshadrinathan et al., 2010). Specifically, we considered 1.5 · 10^5^ randomly-selected patches of spatial size 16 × 16 and 32 temporal frames. These patches were analyzed with the set of linear spatio-temporal sensors described in Section 1.1 that models the linear part of motion sensitive V1 (Heeger, 1987; Simoncelli and Heeger, 1998).

For a better understanding of the static motion aftereffect, we focus on the statistics of the responses of a selected subset of this filter bank. As illustrated in Figure 3, misinterpretation of the motion of a static noise pattern after adaptation to moving stimuli is due to the inhibition of the sensors tuned to static stimuli (*f*_*t*_ = 0) caused by the high activity of neighbor sensors of the same spatial frequency but different temporal frequency. This inhibition (smaller response due to greater activity of the neighbors as in Figure 2) induces the imbalance of the response pattern and the incorrect decoding of speed.

The statistical derivation of the different operation regimes from natural (complex) scenes implies specific assumptions about how the adaptation environment and the test environment are constructed. As a result, the comparison with the experimental literature on gain control, e.g., adaptation and masking (Ross and Speed, 1991; Foley and Chen, 1997), is possible but not straightforward.

In classical experiments, one chooses which stimuli are presented before the test (adaptors) and which stimuli are presented as a pedestal for the test (masks). Therefore, adaptation and masking are distinct paradigms to study the effect of the context on the visibility of a test. In the statistical case, when gathering the samples to build the training sets one could take a literal approach: look for the required change of environment in the database and sample only from those cases (e.g., look for situations in which a certain motion is suddenly followed by a static scene). The problem with this literal approach is that it is difficult to find enough samples with the required conditions for a reliable estimation of the responses. For instance, such sudden motion changes are rare in nature.

In order to simplify obtaining the training sets, we define the adaptor and the test environments in a slightly different way, which determines the kind of gain changes these training sets can represent. We take samples disregarding which one comes first in a temporal sequence. Then, if we are interested in the response of a particular sensor tuned to a certain test, our set for the adapting environment consists of complex patches in which other linear sensors are *simultaneously* active, e.g., because of a particular motion. Our set for the test environment consists of simpler patches in which only the sensor tuned to the test is active (the other patterns in the adaptor environment are not present in the test environment).

As a result, we build the adaptors for aftereffects from natural environments where simultaneous stimulation of multiple sensors occur, i.e., similarly to classical masking. This adapting environment made of complex stimuli should reduce the response and lead to aftereffects since the regime in the test environment is different. In the following description (both in this motion experiment and in the texture experiment below), the stimulation in coefficients adjacent to the test in the adapting environment will be denoted using the *adap* subindex.

The relevant statistics for the waterfall effect involve the joint distribution of responses of linear filters tuned to zero temporal frequency and the corresponding neighbors tuned to progressively different temporal frequencies (or speeds). Accordingly, we choose the illustrative case of the filter tuned to (*f_x_*, *f*_*y*_, *f*_*t*_) = (0 *cpd*, 10.8 *cpd*, 0 *Hz*), relevant for vertical motion illusion. In order to stress the generality of the result (as in the examples of Figure 1), we also consider a different orientation: the filter (*f_x_*, *f*_*y*_, *f*_*t*_) = (10.8 *cpd*, 0 *cpd*, 0 *Hz*), for horizontal motion illusion. We will consider the response of the above two filters in the presence (or absence) of activity in other sensors of the same spatial frequency, but different temporal frequencies in the range *f*_*t*_ = [−9 *Hz*, +9 *Hz*]. According to the equation of the optical flow (Watson and Ahumada, 1985), $\vec{f}\cdot \vec{v}+f_{t}=0$, the above frequency range implies that in this illustration we consider the response of sensors tuned to static patterns as a function of the activity of sensors tuned to (vertical or horizontal) speeds in the range *v* = [−0.83°∕*s*, +0.83°∕*s*].

**Figure 4:**
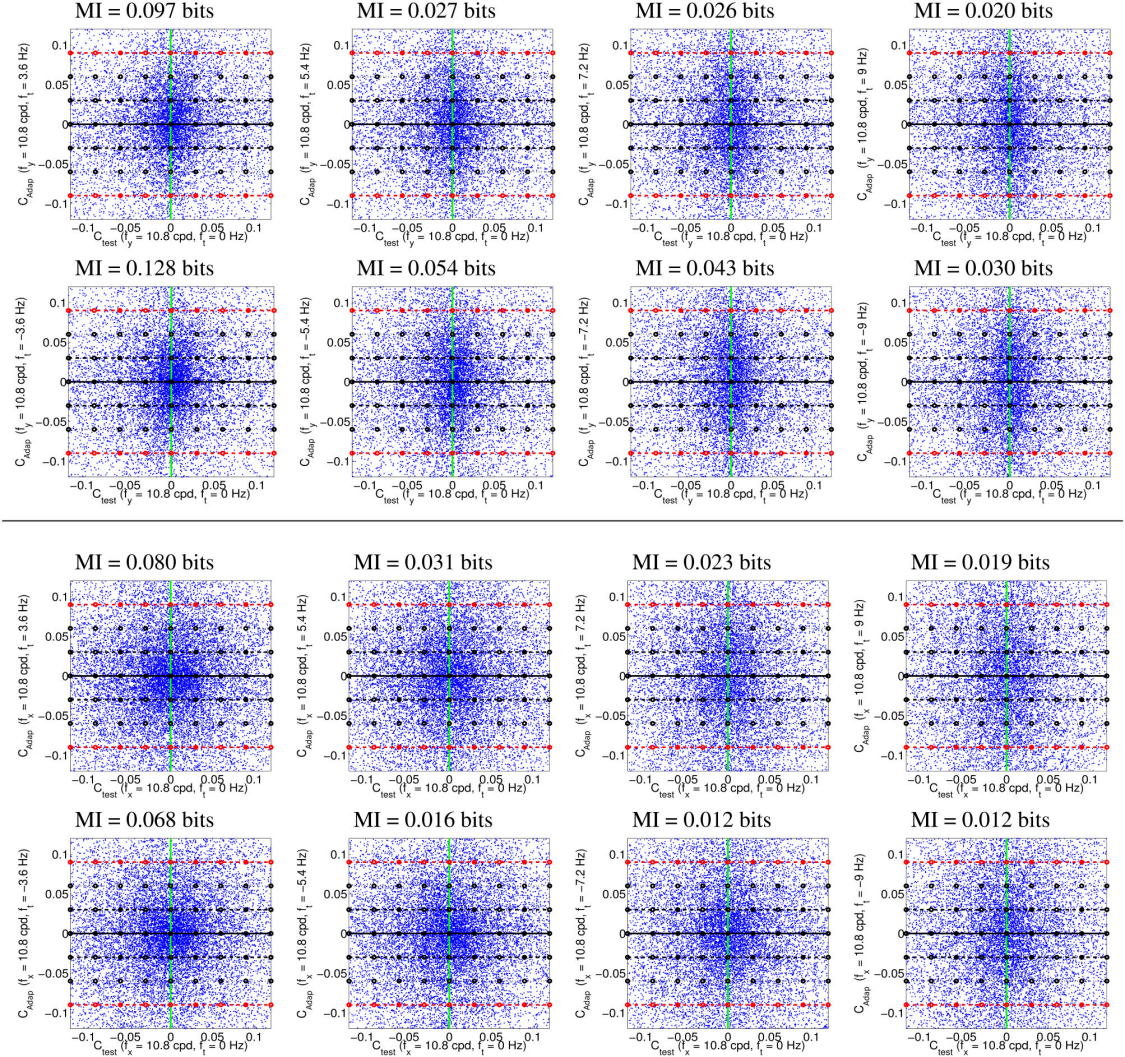
**Natural movie data after the linear stage**. Scatter plot of the responses to natural sequences of V1-like linear motion filters tuned to zero speed (*C*_*test*_ in abscissas), and to different non-zero speeds (*C*_*adap*_ in ordinates). **Top** (1st and 2nd rows) shows relevant data for the vertical motion aftereffect: linear responses of sensors in the plane (0, *f*_*y*_, *f*_*t*_). **Bottom** (3rd and 4th rows) shows relevant data for the horizontal motion aftereffect: linear responses of sensors in the plane (*f_x_*, 0, *f*_*t*_). First and second rows in each panel represent the interaction of the *f*_*t*_ = 0 sensor with sensors of positive and negative speeds. In each row, the modulus of the speed of the considered adaptor increases from left to right: from close sensors to very distant sensors. The mutual information numbers (MI in bits) above each plot show that the relation between responses decreases as the distance in frequency increases. As in Section 1.1, the magnitude of the stimulation is expressed in *contrast* (amplitude over standard deviation of the response).

Figure 4 illustrates the uneven distribution of natural sequences in different subspaces of the linear response domain: each blue dot represents the responses elicited by a video sample in the two sensors considered. In every case, the abscissas correspond to the stimulation (contrast) of linear sensors tuned to static stimuli (*f*_*t*_ = 0). The green line represents zero contrast for the *f*_*t*_ = 0 sensor. Displacements away from the green line along the abscissa (represented by the horizontal lines in different styles) represent non-zero contrast for the *f*_*t*_ = 0 sensor. Positive and negative displacements along the abscissa only differ in the stimulus phase but are otherwise equivalent: higher stimulation (higher contrast) for greater distance from the green line. The ordinate axis has the same interpretation (higher stimulation for greater distance from the black solid line) but for different sensors. The different plots along the rows correspond to sensors in the ordinate axis with *f*_*t*_ ≠ 0, where |*f*_*t*_| progressively increases from left to right: 2-*d* subspaces spanned by close or distant sensors. The top rows correspond to positive speeds and the bottom rows to negative speeds in the corresponding neighbor sensor.

We denote the abscissa and ordinate axes as *test* and *adap*, respectively since we are interested in the response to static stimuli (test) in different adapting environments defined by the presence of other moving stimuli. The black solid line, *C*_*adap*_ = 0, represents stimulation of the *f*_*t*_ = 0 sensor for zero activity of the other sensor (isolated static stimuli). The other horizontal lines, *C*_*adap*_ ≠ 0, represent stimulation of the *f*_*t*_ = 0 sensor in an environment where other moving stimuli are present. Horizontal lines with the same |*C*_*adap*_| above and below the black solid line represent equivalent environments where the moving adaptor has the same contrast and speed but a different phase. Positive and negative responses come from phase coincidence or opponency between filter and stimulus. Since having one phase or its opposite is equally likely in small patches from natural scenes, different signs are equally frequent. As a result, the scatter plots are symmetrical with regard to the origin.

The scatter plots in Figure 4 suggest a different interaction for close and distant frequencies. Qualitatively speaking, the scatter plot is *more rounded* for closer frequencies (left) and *more elongated* for distant frequencies (right). As a result, the conditional probabilities *P*(*C*_*test*_|*C*_*adap*_) clearly depend on the contrast *C*_*adap*_ for closer frequencies, while they are more independent for distant frequencies. Greater statistical dependence between the responses of closer filters is quantitatively confirmed by the mutual information numbers (MI in bits) shown above each plot: see how the MI decreases from left to right.

Note that these general statistical trends are the same for positive and negative phases: scatter plots are roughly symmetric with regard to the green line. The distribution of responses for different phases of the adaptor is also the same: symmetry with regard to the solid black line. Positive and negative speeds share similar properties too: the first and second rows in each panel are similar. This indicates that upward motion has the same distribution as downward motion. And the same equivalence holds for left/right motion.

#### 2.1.2. Nonlinear responses to moving patterns from sequential principal curves analysis

We use the responses of linear sensors to natural sequences as a set to train optimal nonlinear sensors according to biologically sensible organization criteria: (1) information maximization -*infomax*-, and (2) error minimization. In the methodological Section 4, we explicitly show how SPCA does this job for visual textures by changing its metric parameter. For now, one merely has to know that, similarly to other unsupervised learning algorithms (such as PCA or ICA), SPCA identifies directions (curves) in the data space. These directions can be interpreted as sensors, or axes of a representation system. The response of these sensors is given by the projection of the data onto the representation axes. As discussed in more detail in Section 3, the advantage of SPCA with regard to linear techniques is that (i) the identified directions may be curves instead of straight lines, and (ii) the line element (or metric) in these curves may be non-uniform, which is equivalent to a nonlinear response.

**Figure 5:**
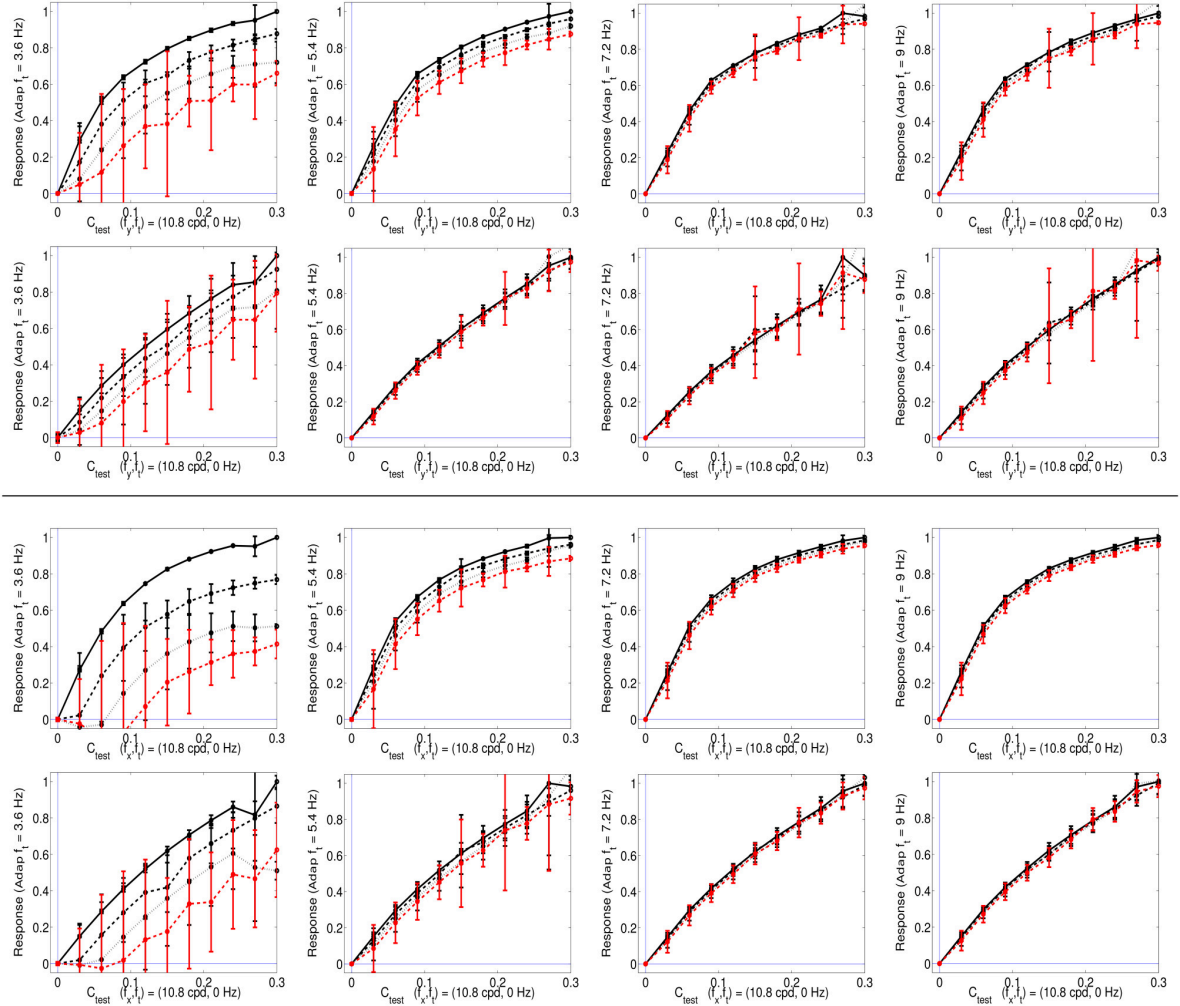
**Responses of SPCA sensors tuned to ***f***_***t***_ = 0 in environments where other moving patterns are present**. **Top** (1st and 2nd rows) responses with adaptation to *vertical* speeds. First row displays the response of SPCA tuned for infomax while second row shows the equivalent results for SPCA sensors with minimum representation error. In every row, the vertical speed of the adapting stimulus increases from left to right. As in Figures 2, 4, different line styles correspond to the responses in different environments: the solid black line corresponds to responses to isolated static stimuli, and the other line styles correspond to responses of the same sensor in environments where there is also a moving pattern of progressively greater contrast: from the dashed-black line (moving adaptor with small contrast), to the dashed-red line (moving adaptor with greater contrast). The curves are the average over equivalent stimulations (phase and speed sign). Error bars indicate standard deviation. **Bottom** (3rd and 4th rows) equivalent responses with adaptation to *horizontal* speeds.

Here we apply SPCA to the training data in Figure 4 using the aforementioned organization principles. In particular, we are interested in the response of the SPCA sensor tuned to zero speed since its eventual attenuation is what explains the static motion after effect (see Section 1.1). When training SPCA on the above 2-*d* spaces, it identifies two principal curves, or two sensors. Figure 5 show the responses of the SPCA sensor more aligned with the horizontal axis in Figure 4. In particular, we computed the second-stage response of such sensor (projections onto that curve) for the data (first-stage responses) along the highlighted horizontal trajectories in Figure 4. As discussed above, moving along these lines means increasing the contrast of the static pattern in different environments: in isolation (black solid line), or in the presence of other (moving) patterns with different contrast (progressively greater contrast from the black dashed line to the red line). In this computation we averaged the SPCA responses over equivalent conditions (different phase and speed sign), hence the standard deviation bars.

As seen in Figure 5, the response of the second-stage sensor turns out to be nonlinear with the contrast of the test, and more importantly, it is strongly attenuated by the presence of high contrast moving patterns with low temporal frequency (see plots on the left). When the initial moving pattern has a more distant temporal frequency its effect on the response is negligible (plots on the right). This different influence as a function of the distance between the frequencies of test and adaptor actually comes from the *rounded-to-elongated* trend in the scatter plots discussed in Figure 4. SPCA only derives the optimal second-stage responses for these signals and captures the trend of the data after the linear stage.

This statistically-derived behavior is similar to the empirical description in Section 1.1, and reproduces the basic trends in Morgan et al. (2006), Mather et al. (2008), and Stocker and Simoncelli (2009) regarding the decrease of the motion aftereffect with the distance in temporal frequency. The different design strategies lead to different saturation rates (sharper nonlinearity for infomax), but similar contrast-dependent attenuation for close temporal frequencies, which is key for the aftereffect.

### 2.2. Texture aftereffect from SPCA

#### 2.2.1. Visual texture data after the linear stage

**Figure 6:**
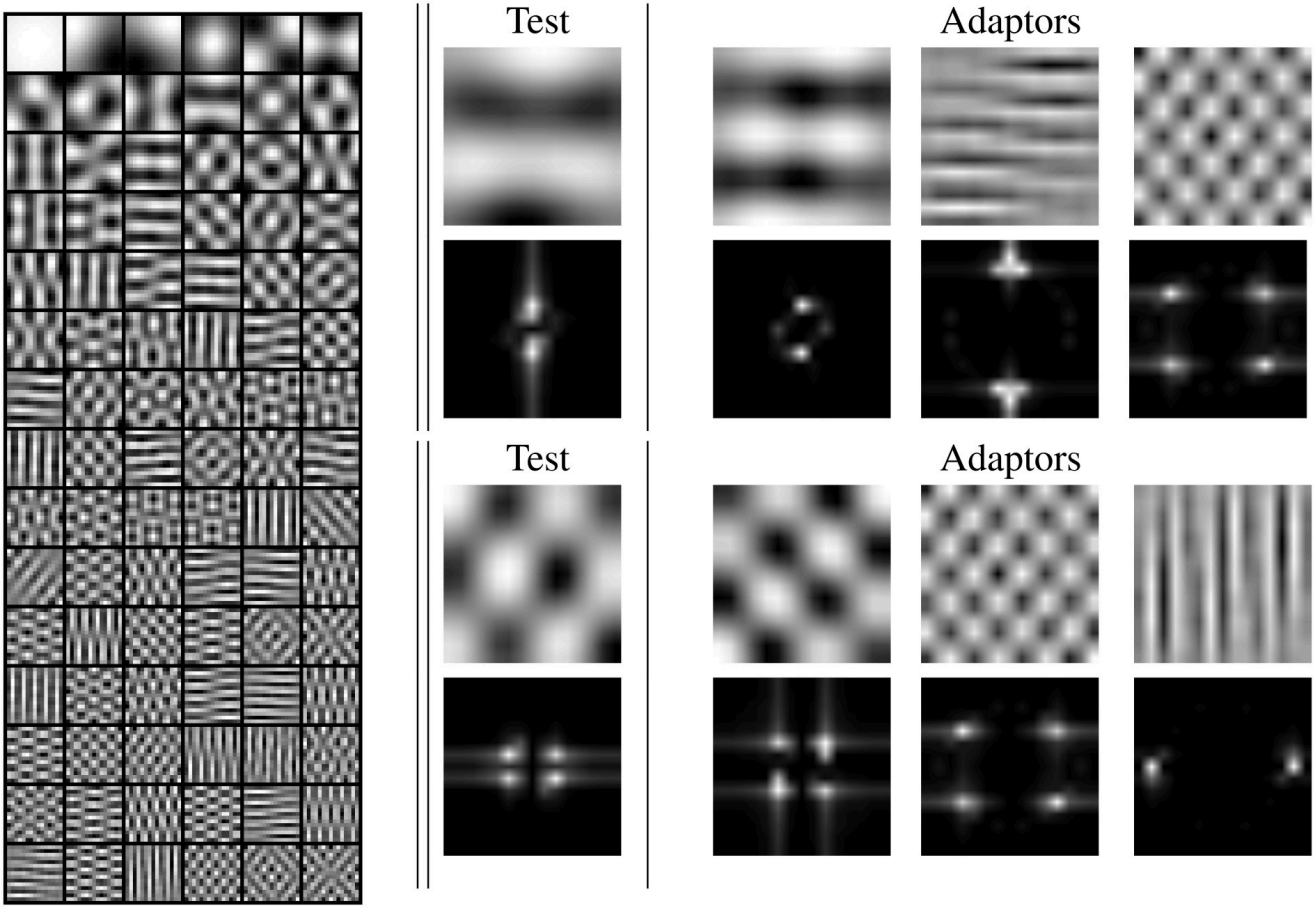
**Left** linear receptive fields from natural textures using PCA. **Center** *test* stimuli (or selected filters) in the spatial domain (oscillatory patterns) and in the frequency domain (narrow band light regions on dark background). **Right** stimuli to set different adaptation environments (adaptors) for the test on the center panel. The Fourier transforms show the frequency similarity between *tests* and *adaptors*.

We studied the statistics of natural visual textures by gathering luminance samples from the McGill natural image database (Olmos and Kingdom, 2004). Specifically, we randomly selected 1 · 10^6^ patches of spatial size 15 × 15, and analyzed their distribution after the linear stage. *L* was simulated using a simple 2nd order approach: PCA-based whitening (Hancock et al., 1992; Olshausen and Field, 1996). Figure 6 (left) shows the PCA feature extractors (or linear receptive fields) that capture most of the energy of the texture samples considered.

We focus on the statistics of the response of a specific linear sensor when stimulated in different environments (when other specific linear sensors are active). We consider two examples of low frequency first-stage sensors denoted as *test* in the central panel of Figure 6. Their orientation (e.g., horizontal or diagonal) does not affect the basic trend. Following the experimental literature (Foley, 1994; Foley and Chen, 1997; Watson and Solomon, 1997), for each sensor, we consider three different environments inducing activation in neighbor sensors with: (1) similar frequency and orientation, (2) different frequency but similar orientation, and (3) different frequency and orientation. Note that textures in Figure 1 are built using these PCA functions. In each case, we consider what we call the *no-adaptation condition*, in which the test is shown on a zero contrast background, and progressively stronger *adapting conditions*, in which the test is shown on top of an adaptor with progressively higher contrast.

**Figure 7:**
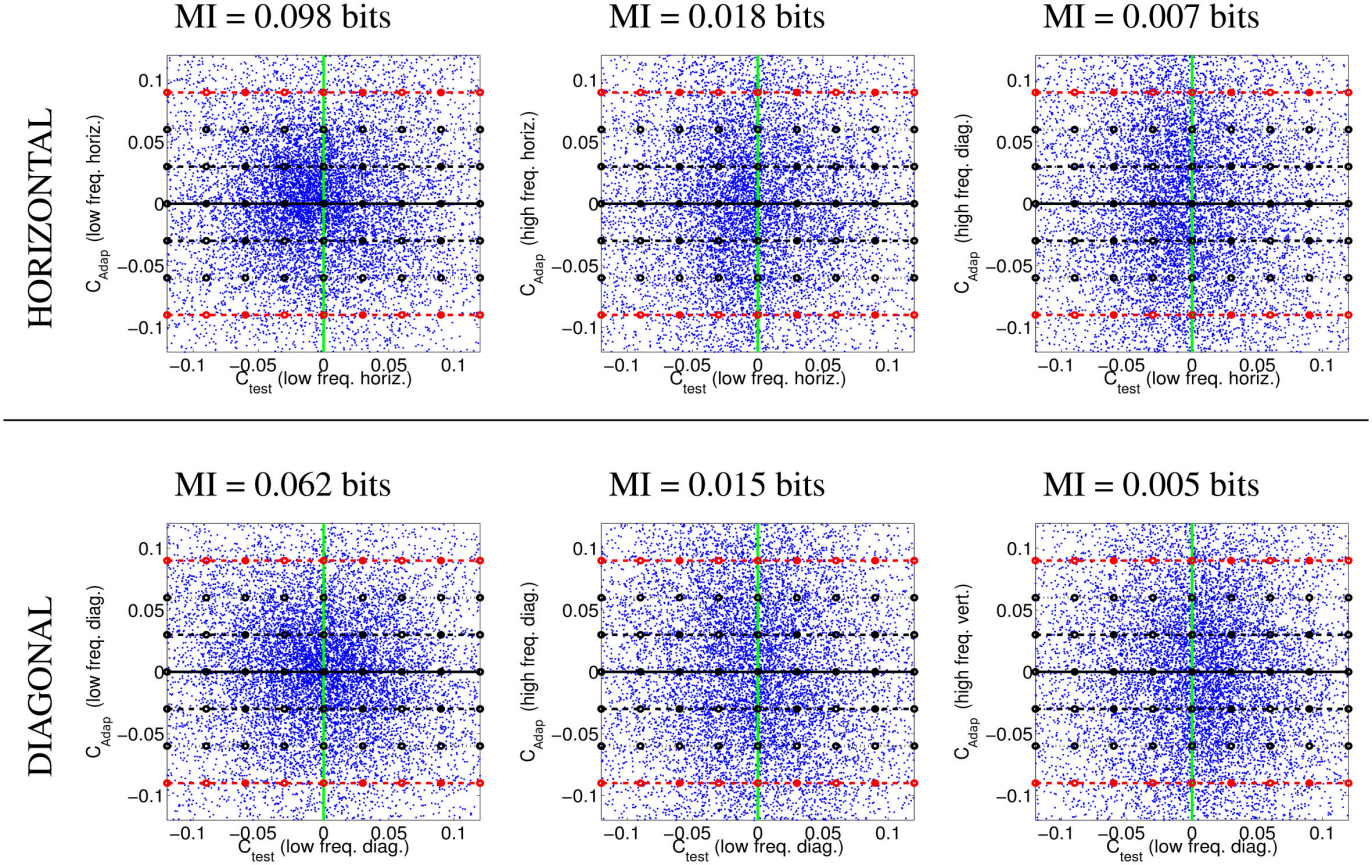
**Natural visual textures after the linear stage**. Image patches projected on the axes defined by the linear receptive fields selected to describe the different adapting environments. **Top row:** projections on the low frequency horizontal analyzer and different additional analyzers: low frequency horizontal (left), high frequency horizontal (center), and high frequency diagonal (right). **Bottom row:** projections on the low frequency diagonal analyzer and different additional analyzers: low frequency diagonal (left), high frequency diagonal (center), and high frequency vertical (right). The mutual information numbers (MI in bits) above each plot show that the relation between linear responses decreases as the distance in frequency increases. As in the motion experiments, the magnitude of the linear responses are expressed in *contrast* (amplitude over standard deviation).

Figure 7 shows scatter plots of projections of natural image patches on the directions of the considered linear pattern analyzers discussed above. Figure 7 shows the subset of natural patches with low amplitude in all the other dimensions: we considered natural textures living in the 3-*d* subspaces formed by the DC component and the two components shown in the scatter plots. This condition is satisfied by about 1.5 · 10^5^ samples in each 3-*d* subspace. As in Figure 4, horizontal lines in different styles in Figure 7 represent stimulation of the considered low-frequency linear sensor (horizontal, top; or diagonal, bottom) in different environments with progressively higher contrast in the adapting environment. From left to right, the adapting stimulus (or neighbor linear filter) considered in the ordinate axis goes from similar frequency and orientation to very different frequency and orientation (they correspond to the *adaptors* in Figure 6).

The scatter plots show the behavior of natural textures. For similar frequencies the conditional PDFs *P*(*C*_*test*_|*C*_*adap*_) clearly depend on the value of the environment *C*_*adap*_. However, for very different frequencies and orientation, the conditional PDFs are relatively more independent of *C*_*adap*_: note that the distribution in the case of very different frequencies is *more elongated* in the vertical dimension. That is *C*_*test*_ and *C*_*adap*_ are more statistically independent when their frequencies are far away from each other. The mutual information values confirm this intuition from the scatter plots. This trend is consistent with previously reported results on dependence between image transform coefficients (Simoncelli, 1997; Buccigrossi and Simoncelli, 1999; Hyvärinen et al., 2003; Gutiérrez et al., 2006; Malo et al., 2006; Camps et al., 2008; Hyvarinen et al., 2009; Malo and Laparra, 2010).

#### 2.2.2. Nonlinear responses to texture from sequential principal curves analysis

**Figure 8:**
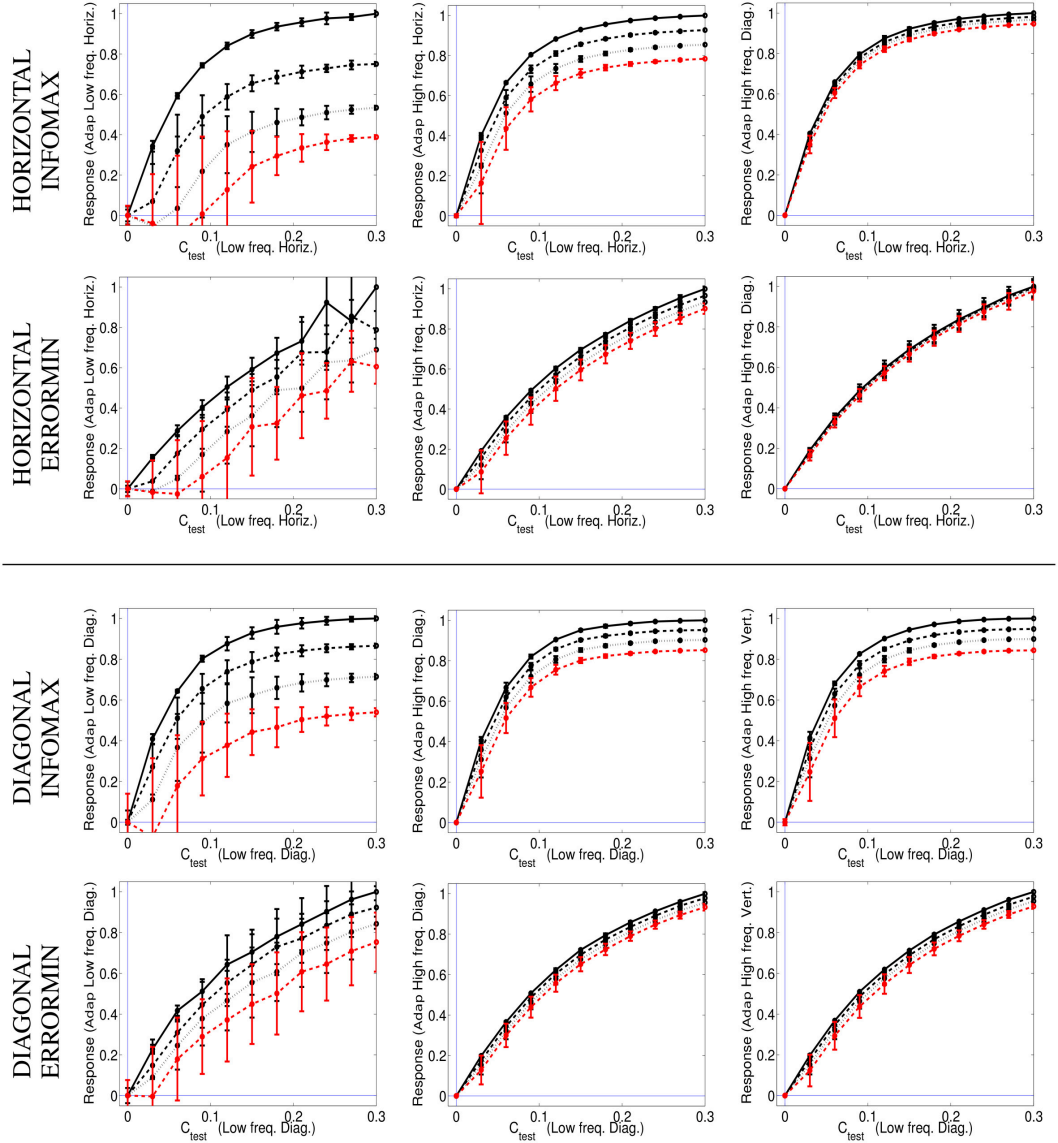
**Responses of SPCA texture sensors in environments where other textures are present**. **Top** (1st and 2nd rows) responses of the SPCA low frequency *horizontal* sensor. These nonlinear responses correspond to the training data in the top row of Figure 7. **Bottom** (3rd and 4th rows) responses of the SPCA low frequency *diagonal* sensor. These nonlinear responses correspond to the training data in the bottom row of Figure 7. In each panel, two design strategies were used (infomax, top, and error minimization, bottom). As discussed in Figures 6, 7, the frequency distance between test and adaptor increases from left to right. Responses are the average of the realizations of equivalent stimulation conditions. Since stimulation (and adaptation) with positive or negative contrast is equivalent (note the symmetry of the scatter plots), the responses shown are the average over these equivalent observation conditions. Different line styles correspond to progressively stronger contrast of the adapting environment (as in the scatter plots in Figure 7).

Figure 8 shows the responses of the selected SPCA sensors (low frequency horizontal and low frequency diagonal, respectively) in different environments (adaptation conditions) and using different SPCA metrics (infomax or error minimization). As in the motion section, results are the average of the responses over equivalent conditions (different phases of test and environment), hence the standard deviation bars.

The results show the emergence of different operation regimes depending on the environment. When presenting the test on top of backgrounds of similar frequency the nonlinear response is strongly attenuated as the background contrast increases. In contrast, the nonlinear response is not severely affected when the test is shown on top of backgrounds that significantly differ in frequency (either in modulus or orientation). Optimal SPCA sensors capture the differences with distance in frequency suggested by the distribution of natural signals in the scatter plots.

SPCA behavior (Figure 8) is consistent with the behavior of V1 sensors (Carandini and Heeger, 1994; Cavanaugh et al., 2002; Carandini and Heeger, 2012). Therefore, SPCA reveals that the saturating nonlinearity and the effect of different environments have strong statistical grounds: both *infomax* and *error minimization* strategies give rise to these *qualitative* trends.

The different design strategies lead to different saturation rates (sharper nonlinearity for infomax, top row in Figure 8), but similar contrast-dependent attenuation for close spatial frequencies, which is the relevant feature to explain the texture aftereffect. Existence of different response regimes as a function of the environment implies that after seeing a high contrast pattern of certain frequency content, frequency analyzers in that spatial region will be attenuated. As a result, contrast of stimuli of similar frequency and orientation seems to fade out in that region despite the (physical) stationarity of the stimuli.

### 2.3. Color aftereffect from SPCA

#### 2.3.1. Color data from a biased reflectance world

SPCA was applied on the IPL database in Laparra et al. (2012) to statistically explain the nonlinear responses of the chromatic channels and the reported shift in the Red-Green channel under illumination change from CIE D65 to CIE A (Krauskopf and Gegenfurtner, 1992). As stated in Section 1.1, this behavior leads to color illusions after illumination changes. As suggested, but not addressed, in Abrams et al. (2007) and Laparra et al. (2012), similar shifts in the operation regime (and hence equivalent aftereffects) should happen when changing the statistics of the reflectance of the environment instead of changing the illuminant. This is the statistical experiment we address in this section.

**Figure 9:**
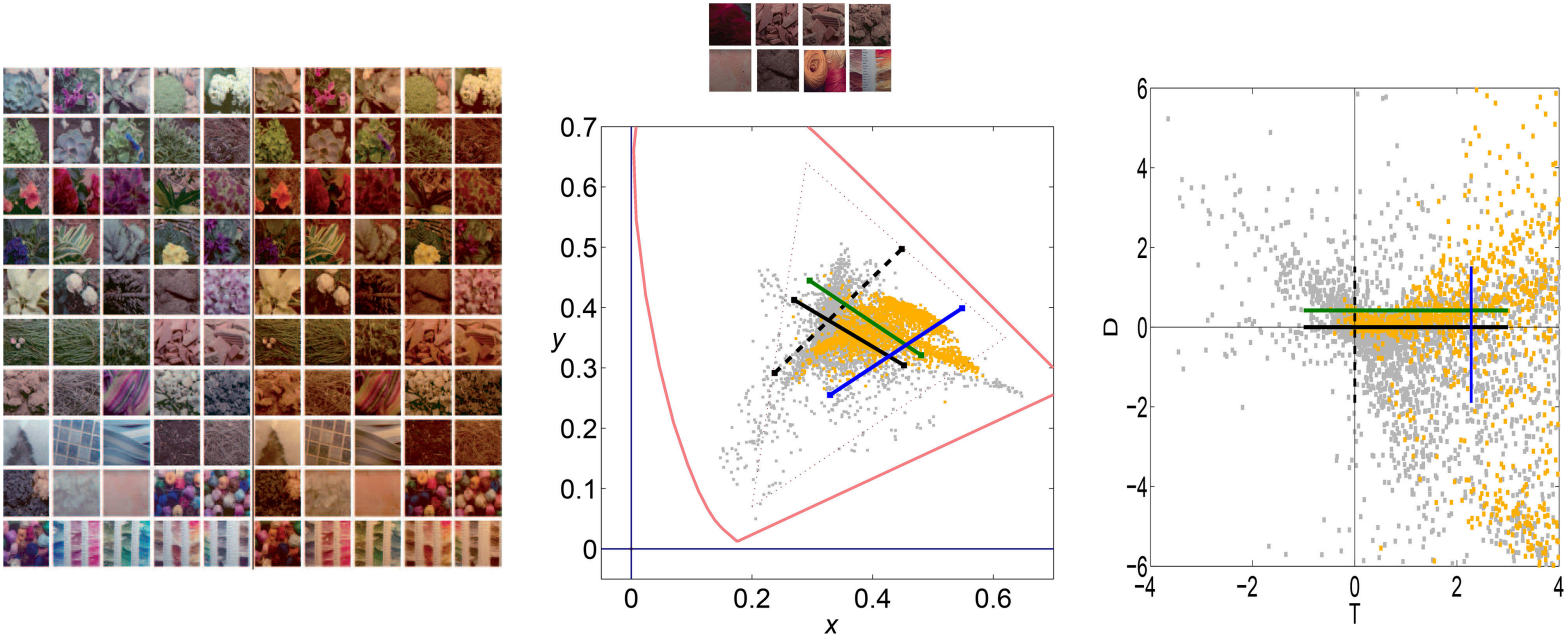
**Color datasets coming from balanced and biased reflectance sets**. The large panel of color images (on the **left**) shows a *balanced* database consisting of a diversity of objects under CIE D65 and CIE A illuminants (Laparra et al., 2012; Gutmann et al., 2014). The colors under D65 are plotted in gray in the CIExy chromatic diagram in the center. The small panel of colored images at the top-center were selected from the D65 illumination dataset, but constitute a *consistently biased* world with restricted reflectance (yellow dots in the chromatic diagram). Note that the restricted set is biased to red both in the CIExy diagram **(center)** and in the linear Ingling and Tsou space **(right)**. Black lines represent the stimulation of the T or D sensors (in solid and dashed style, respectively) in a situation in which the other sensor gives no signal (appropriate for an average gray world). Colored lines represent the stimulation of the T or D linear sensor (in green and blue, respectively) in a situation in which the T and D linear sensors have positive response (a red-yellowish world).

Figure 9 shows color sets corresponding to (1) a natural world with a variety of surfaces of different reflectance under CIE D65 illuminant (left), and (2) a restricted world consisting of reddish objects only (biased reflectance) but under the same illuminant (middle-top). This setting would correspond to situations like the example with natural colored images in Figure 1 (from the IPL database). Here we assume the linear stage is a transform to a psychophysically sensible opponent color space (Ingling and Tsou, 1977) which is similar to a decorrelation transform. The chromatic channels are Red-Green (or T for *trinanopic*) and Yellow-Blue (or D for *deuteranopic*). Figure 9 (right) shows the linear responses to the objects of the two scenes considered (balanced vs. biased) in the chromatic TD plane.

Lines in the TD plane and the CIExy diagram represent the stimulation of the T and D sensors in different environments: (1) the black lines represent the stimulation of each sensor with no stimulation in the other sensor, and (2) the colored lines represent equivalent stimulation in an environment where the other linear sensor has certain average response or bias (e.g., the reddish environment).

#### 2.3.2. Nonlinear responses to color from SPCA

**Figure 10:**
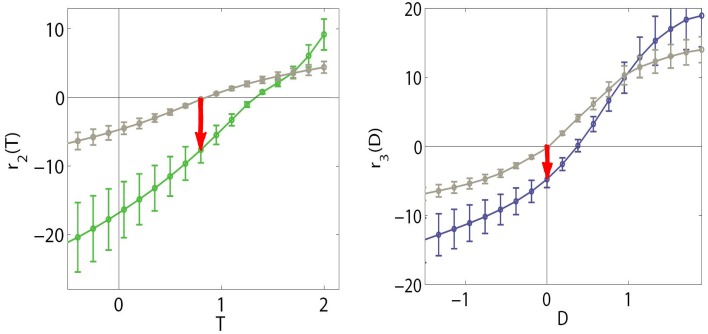
**Shifts in the nonlinear response of SPCA color sensors when adapted to biased image statistics**. When adapted to the biased environment, the responses of the sensors identified by our method (RG in green on the left and YB in blue on the right) shift with regard to the curves in gray (adaptation to a diverse environment, large D65 database and stimulation along the black lines in Figure 9). Note that in this case, a stimulus eliciting zero responses (perceived as white) in the diverse environment situation, would elicit negative responses in the RG and YB mechanisms (red arrows) in the restricted environment.

We computed the response of SPCA sensors along the stimulation lines depicted in Figure 9 using the color data from the environments of different chromatic natures (balanced vs. biased reflectance set). Figure 10 shows that different operation regimes were found in the RG and YB mechanisms in the different environments: the response curves (and what is considered to be the origin) shift. Figure 10 only shows the SPCA responses for the error minimization criterion since they better represent the experimental behavior of the color sensors (Laparra et al., 2012). Nevertheless, as in the motion and texture cases, information maximization results (not shown for color) give rise to qualitatively similar shifts but sharper nonlinearities. This difference is clear from the relation between data density and the strategy-dependent metric in SPCA (see the technical Section 4), and it is consistent with previous comparisons of infomax and error minimization strategies (Twer and MacLeod, 2001; MacLeod and von der Twer, 2001; MacLeod, 2003).

Locations in the nonlinear opponent representation space are decoded as follows. A stimulus leading to zero response in both nonlinear chromatic mechanisms is interpreted as *white*. A positive response in the nonlinear T channel is decoded as *red*, while a negative response is perceived as *green*. A positive response in the nonlinear D channel is decoded as *yellow* and a negative one is decoded as *blue*.

In the *diverse reflectance* world, the origin of the chromatic plane corresponds to the values where the responses of both chromatic mechanisms (gray curves in Figure 10) is zero. Restriction of the set of objects available in the environment implies a shift in the training data (linear responses) and, as a result, a change in the location of the origin in the nonlinear response space. Given the shift in the SPCA response curves, after the adaptation to reddish objects, an object that would be interpreted as white in the diverse world (gray curves) elicits negative responses in the sensors (see red arrows to the colored curves). Therefore, it would be perceived as blueish-greenish, as is the case in human observers undergoing a sudden change of environment (surface statistics). This explains the general trend of the aftereffect.

## 3. Discussion

### 3.1. Summary: changes in the statistics explain the aftereffects

Results in Section 2 show that the proposed analysis of the scene statistics reproduces the trends of the considered illusions. Optimal SPCA sensors derived from natural data of different modalities (motion, texture, color) have characteristic nonlinear responses that resemble those of the biological sensors. Moreover, these responses display different environment-driven operation regimes (Figures 5, 8, 10) that explain the aftereffects. These distinct regimes come from the structure of natural signals, for example (1) the shift of color distribution in specific environments (e.g., with restricted hue), and (2) the dependence or independence of the linear frequency responses as a function of the environment (e.g., the presence of a certain pattern or moving stimulus). SPCA just captures these statistical regularities in natural data.

The set of statistically-derived responses reported here is compatible with the experimental behavior (response curves or incremental thresholds) found in the different mechanisms. For example, motion sensitive mechanisms display such contrast-dependent nonlinear behavior (Simoncelli and Heeger, 1998) and the incremental thresholds increase when the frequency of the adaptor is closer to the frequency of the test (Morgan et al., 2006), which is equivalent of a greater attenuation for closer frequencies. The same is true for the contrast response of the visual texture mechanisms (Foley, 1994; Watson and Solomon, 1997; Cavanaugh et al., 2002; Carandini and Heeger, 2012). In the above empirical descriptions that use explicit functional forms such as divisive normalization this frequency dependence is related to the extent of the interaction kernel in the denominator (*H* in Equation 2). In contrast, in our non-parametric case, this attenuation comes from the frequency extent of the statistical dependence in the data samples. For the color mechanisms, the current empirical appearance models (Fairchild, 2005) lead to such shifts in the nonlinear responses when using white information from the average color of the scene, and the same is true for models that use explicit functional forms for the nonlinearity (Hillis and Brainard, 2005; Abrams et al., 2007). In our case, SPCA color sensors just follow the data distributions.

As opposed to the empirical descriptions, the different operation regimes found in the different environments using (the non-parametric) SPCA provide a statistical explanation of the considered motion, texture, and color behavior. In the motion perception case, the optimal behavior in an environment dominated by the presence of a high contrast moving pattern (situation 1) implies the attenuation of the neighbor sensors tuned to static stimuli (Figure 5). In environments where these high contrast moving patterns are not present (situation 2) the optimal response of *f*_*t*_ = 0 sensors is greater (they are not attenuated). Similar behavior is statistically found for mechanisms sensitive to spatial texture (Figure 8): in presence of high contrast adaptors (situation 1) the response of sensors tuned to similar frequencies is reduced, while in absence of such adaptors (situation 2) the response is greater. The similarity between the signal statistics in the cases of motion and texture (Figures 4, 7), and, as a result, the similarity in the SPCA nonlinear responses (Figures 5, 8) is consistent with the parallelism that has been reported between motion and texture aftereffects (Clifford, 2002), as well as the similarities in the actual nonlinear responses of the motion and texture mechanisms (Foley, 1994; Morgan et al., 2006). In the case of color vision, reddish environments (situation 1) imply that the response curves of the T and D sensors is shifted to the right (Figure 10) with regard to environments with diverse reflectance (situation 2).

As a result of the different operation regimes, identified by SPCA in different contexts, illusions arise:

- When the moving pattern stops, the new set of responses is unbalanced, and the set of responses is interpreted as coming from a pattern with reverse motion. The illusion is greater for smaller differences in temporal frequencies between test and adaptor, consistently with the psychophysics (Morgan et al., 2006; Mather et al., 2008; Stocker and Simoncelli, 2009), or the example in Figure 1. Of course, we don't claim to reproduce *all* the wide phenomenology of the motion aftereffect. For instance, in Stocker and Simoncelli (2009) the authors were forced to include a two stage adaptation model to accommodate all the experimental details. Here we only focus on the most relevant factor (according to the Stoker et al. results): the de-sensitivity around the adaptor.
- When the high contrast texture disappears, sensors in that area are still attenuated and hence they produce a perceptual hole in the new (physically stationary) scene. The effect is greater if the test and the adapting stimuli have similar frequency content, which is consistent with the classical reports of the illusion (Blakemore and Campbell, 1969; Barlow, 1990), or the example in Figure 1.
- When the reddish world is substituted by a diverse world, stimuli considered to be white in regular situations appear as blueish-green because the nonlinearities are still those optimal for situation 1. This is consistent with human perception (Loomis, 1972; Zaidi et al., 2012), with the descriptions given by empirical color appearance models (Fairchild, 2005), and with the example in Figure 1.

These results suggest that the illusions (apparently dysfunctional behavior) actually come from sensors that try to optimize their response in each (statistical) environment. After a sudden change in the statistics, illusions appear while the sensors change from one optimal operation regime to the other, and disappear once the new optimal reference frame is set.

### 3.2. Related work

Even though the efficient coding suggestions (Barlow, 1990; Wainwright, 1999) have inspired a number of authors that tried to relate aftereffects to the environment in different ways (Webster and Mollon, 1997; Clifford et al., 2000; Weiss et al., 2002; Stocker and Simoncelli, 2006; Coen-Cagli et al., 2009; Schwartz et al., 2009; Series et al., 2009), the approach proposed here, with obvious precedents in Malo and Gutiérrez (2006) and Laparra et al. (2012), is different to such literature.

For instance, Clifford et al. (2000) suggest that a number of illusions result from statistically-driven self-calibration, and propose a single *parametrical* framework based on centering and scaling. These mechanisms are consistent with those proposed in the linear color adaptation context (Webster and Mollon, 1997) to match signal mean and covariance in diverse environments. In the language of control theory, centering is a form of additive gain control while scaling is divisive (or multiplicative) in nature, and may be nonlinear (Carandini and Heeger, 1994). Even though Clifford et al. say that their parametric adaptation model is inspired in efficient coding and suggest it may have positive effects from an information theoretic view, they do not fit the parameters with statistical data but with perceptual data.

As reviewed in Clifford et al. (2007) and Schwartz et al. (2007), adaptation-induced aftereffects have both *encoding* and *decoding* aspects. Nevertheless, the key part is the change in encoding, e.g., empirical gain control (Morgan et al., 2006), which is statistically justified in this work. As pointed out above, aftereffects appear when changes in the encoder are still unknown to the decoder (Stocker and Simoncelli, 2006; Series et al., 2009).

Illusions were analyzed from the optimal decoding perspective for the first time in Weiss et al. (2002): misperceptions of motion direction may come from Bayesian inference involving the prior assumed by the observer and the likelihood function related to the noise introduced by the sensors. The likelihood (or noise sensitivity) depends on the slope of the response, i.e., on the encoding. Bayesian decoding implies that repulsive aftereffects (as those considered here) come from the likelihood and not from the prior (Stocker and Simoncelli, 2006). Adaptation modifies the likelihood by changing the slope of the response. While (Stocker and Simoncelli, 2006) stated the relevance of the likelihood for the first time, they did not derive the optimal nonlinearity from scene data (as we do here): they just assumed a convenient variation of the Signal-to-Noise ratio to illustrate their point. Generally speaking (Clifford et al., 2007), adaptation serves to keep the match between the input statistics and the response of the encoder. The environment-dependent response regimes obtained in Section 2 from a non-parametric method are quantitative illustrations of this general statement. Matching to the scene statistics in the texture tilt illusion has been done through parametric image models such as Gaussian Scale Mixture that lead to divisive normalization-like responses (Coen-Cagli et al., 2009; Schwartz et al., 2009). In these cases, the neighborhoods for the normalization depend on the local texture, which explains attractive and repulsive effects. In Series et al. (2009) the authors analytically explore what happens when the decoder still does not know that the encoder changed (e.g., to follow the statistics). The result is that temporary suboptimality (unawareness) of the decoder leads to aftereffects. Series et al. assume parametric models for the change of the encoder, as in Clifford et al. (2000) and Stocker and Simoncelli (2009) for motion, and divisive normalization for contrast (Carandini and Heeger, 1994).

In contrast, in this work we focus on the changes in the encoder and the reasons for those changes to happen (organization principles). We make no a-priori assumption on the way the encoder should change, i.e., we do not impose divisive normalization in any way but obtain the responses from the signal statistics through a flexible technique. We find different operation regimes depending on the environment. Therefore, the outputs of the adapted encoder are misinterpreted by a temporarily unaware decoder.

The explanation of aftereffects we propose here is qualitatively inspired by the manifold equalization idea suggested, but not implemented, in Barlow (1990) and Clifford (2002). Moreover, the idea of a criterion-dependent metric (not only for infomax but also for error minimization) totally comes from the discussion in Twer and MacLeod (2001), MacLeod (2003), and MacLeod and von der Twer (2001). Unfortunately, in these studies, an explicit, truly multidimensional, algorithm to get the optimal set of sensors remained unaddressed: the authors just showed marginal PDFs in predefined one-dimensional axes. Moreover only the nonlinear behavior (but not the adaptation), was addressed in Twer and MacLeod (2001), MacLeod (2003), and MacLeod and von der Twer (2001). The first step toward the SPCA technique was the nonlinear ICA proposed in Malo and Gutiérrez (2006). Nevertheless, that algorithm was not invertible and the cost function was not clearly defined. These problems were solved with SPCA (Laparra et al., 2012), and the nonlinear ICA goal was extended to include error minimization, as illustrated for visual texture data in Section 4.

An interesting and similar work in spirit is Bednar and Miikkulainen (2000) since they address repulsion in texture aftereffect using unsupervised nonparametric learning through a particular Self Organized Map (SOM). From the technical point of view, the reticle of a SOM can be interpreted as a set of nonlinear sensors implementing some sort of nonlinear equalization mapping (as is the case in SPCA). However, as discussed in Laparra et al. (2012), SOMs not only have practical problems in high dimensional scenarios, but, more importantly, the goal function is not as well-defined, and as easy to tune, as in SPCA. On the other hand, from the perception point of view, Bednar and Miikkulainen did not analyze data from different modalities nor derived the nonlinear responses. Therefore, results in Section 2 better stress the similarity between motion, texturem and color and illustrate the generality of the approach.

Finally, the parametric model in Carpenter and Grossberg (1981) is worth mentioning since, as opposed to most of the empirical literature, the authors somehow derive (i.e., explain) the adaptive behavior from a principle. They invoke the efficient use of a depletable transmitter to propose a particular expression for transduction. From this expression they do derive the shift in the response for different light levels assuming certain time constants in the parameters. Even though the proposed expression is not shown to be optimal in terms of transmitter use, one could argue that they derive the adaptation from an efficiency principle. Our approach (nonparametric and applicable to more visual dimensions) rather than only looking at the constraints of the mechanisms (e.g., limited resolution or limited bandwidth), also considers the regularities of the environment. The behavior results from certain constraints *in* a certain environment.

### 3.3. Distinctive features of the proposed approach and open questions

#### 3.3.1. Explanation vs. description

The proposed approach provides a principled explanation of the phenomena, i.e., it derives the behavior from the environment data and well-defined sensor organization strategies. This is a distinctive feature with regard to empirical models fitted to describe the psychophysics. Moreover, the unsupervised and nonparametric nature of the proposed learning technique makes the point stronger: the derived behavior actually comes from the scene properties and not from a-priori assumed models using specific nonlinearities.

#### 3.3.2. Multiple optimization criteria

SPCA easily accommodates different design strategies (not only infomax, but also error minimization). This is interesting since linear transforms driven by redundancy reduction certainly explain a wide range of phenomena (Buchsbaum and Gottschalk, 1983; Atick et al., 1992, 1993; Olshausen and Field, 1996; Hoyer and Hyvärinen, 2000; Simoncelli and Olshausen, 2001; Doi et al., 2003), however, the generality of this organization principle is still in question (Barlow, 2001). It is not only that additional constraints (such as energy cost, Laughlin, 2004, and matching between features, Gutmann et al., 2014) may be relevant as well, but also, statistical independence may not be the better solution to make optimal inferences in squared error terms (MacLeod and von der Twer, 2001; Simoncelli, 2009; Laparra et al., 2012). Therefore, identifying the guiding principles of visual phenomena requires unsupervised learning algorithms that can be tuned to different specific *goals* (and not only to redundancy reduction). In this way one can falsify alternative organization strategies. Note for instance the different sharpness in the predicted responses in Figures 5, 8 depending on the goal. The wider range of criteria in SPCA is an advantage over previous statistical approaches that may be seen as particular cases of SPCA. For instance, mean shift and covariance matching through scaling, as suggested in Webster and Mollon (1997) and Clifford et al. (2000), can be understood as a 2nd-order decorrelation since it can be achieved through conventional PCAs before and after the environment change. As illustrated in Section 4, SPCA generalizes PCA in this redundancy reduction context because it takes higher-order (instead of just 2nd-order) relations into account. Therefore, SPCA may reduce to PCA, and hence to plain decorrelation, by setting the appropriate values in the parameters, but otherwise it is more general. Other linear adaptation techniques may also take higher-order relations into account, e.g., using coupled ICAs or more sophisticated techniques (Gutmann et al., 2014). However, these approaches would have the linearity constraint that prevents complete achievement of the statistical independence goal: again a restricted version of infomax. As stressed in next paragraph SPCA does not have the linearity constraint that may restrict its statistical effect. On the other hand, SPCA may also be optimal in reconstruction error terms, a criterion which is not necessarily related to decorrelation.

#### 3.3.3. Nonlinear vs. linear

Linear learning methods (e.g., manifold matching through centering and scaling, coupled PCAs for decorrelation and matching, or even coupled ICAs for higher order independence) necessarily disregard the nonlinear nature of the visual sensors: they cannot account for the non-uniform discrimination between patterns (Foley and Chen, 1997), sequences (Morgan et al., 2006), or colors (Krauskopf and Gegenfurtner, 1992). Linear methods may explain response mismatches leading to aftereffects, but more flexible approaches like SPCA are required to provide unified explanations of the contextual attenuation in the response and the fact that it is nonlinear in every context. From a normative perspective nonlinearities of the sensors imply that the decorrelation explanation of aftereffects is a simplified view of the goal that the sensory system is actually optimizing.

#### 3.3.4. Nonparametric vs. parametric

Other principled approaches take (already nonlinear) parametric expressions from empirical models, e.g., divisive normalization in (Schwartz and Simoncelli, 2001; Abrams et al., 2007; Series et al., 2009; Lyu, 2011), or consider parametric image models that lead to divisive normalization-like responses as in Coen-Cagli et al. (2009) and Schwartz et al. (2009). While these approaches certainly derive contextual changes of operation regime, they are not actually explaining the nonlinear behavior, but fitting it to the image statistics. The example in Section 1.1 illustrates the fact that assuming a divisive normalization model almost automatically leads to the required response changes even with generic parameters. The nonparametric nature of SPCA implies no prior assumption on the response. Therefore, it is more clear that the attenuation of the nonlinear responses actually come from the scenes and the optimization goal, and not from a prior inspired in the empirical literature.

#### 3.3.5. Invertibility and metric

In order to make experimentally testable predictions, invertibility of the learned transforms and easy computation of discrimination measures in the stimulus space is highly desirable. Invertibility implies that the relevant features for a particular goal can be analyzed in the stimulus domain, where perception experiments operate. The ability to derive discrimination metrics is a fundamental issue since threshold measurement is a major paradigm in psychophysics. Some of the above are unusual properties in the plethora of nonlinear techniques constantly emerging from the machine learning community (Lee and Verleysen, 2007). In contrast, SPCA learns a set of separate nonlinear sensors that define a signal-adaptive curvilinear reference system; it defines discrimination metrics according to different organization goals; and it is invertible.

#### 3.3.6. Temporal issues

Our proposal identifies distinct optimal operation regimes in different environments but, in its current form, it does not address the issue of the time it takes to change from one regime to the other (or the temporal duration of the aftereffect). Temporal scales of adaptation are an important question (Webster, 2011) and sometimes a measure of the aftereffect strength (Mather et al., 2008). Current SPCA cannot address this issue since it is a *batch* algorithm as opposed to *on-line* algorithms that can evolve as they receive new samples (Lee and Verleysen, 2007). Future on-line algorithms to explain aftereffects should find a balance between the learning rate (speed in updating the reference system) and the incoming information rate. This learning rate is probably mediated by a robustness/fidelity balance. What is the optimal balance in the visual mechanisms is an interesting open question.

#### 3.3.7. Coding-only approach

Another limitation of the proposed technique is that it is focused on the coding part of the coding/decoding problem. In fact, taking into account mismatches or delays between coder and decoder (Series et al., 2009) is other approach to the temporal issues. Nevertheless, it is important to note that while the likelihood function (or the encoding part) is the one responsible for the repulsive aftereffects (Stocker and Simoncelli, 2006), it is commonly modeled using parametric descriptions (Clifford et al., 2000; Series et al., 2009; Stocker and Simoncelli, 2009), and here we investigate *why* the encoder behaves in that way.

#### 3.3.8. Abstract mechanisms

Normative explanations do not intend to describe how actual mechanisms work (Dayan and Abbott, 2005). Therefore, we do not claim the physiological plausibility of SPCA sensors: empirical models reviewed in Section 1.1 provide more realistic descriptions. In contrast, SPCA has to be understood as a normative explanation that builds *abstract mechanisms* from well-defined goals. This derivation of the perceptual behavior from statistical data is a quantitative evaluation (not a description) of the visual system's adaptation mechanisms. In fact, in the construction of SPCA responses a number of physiologically arguable approximations are done. The first is additivity of the differential behavior. In Capilla et al. (1998) there are some comments on the integrability of differential spaces such as Derrington et al. (1984). Another oversimplification is the consideration of a single linear+nonlinear layer. Deep (multi-layer) neural networks, which may be trained for information maximization too (Laparra et al., 2011), may be the way to go. In particular, Malo and Simoncelli (2015) have shown that psychophysically meaningful multi-layer models reduce statistical redundancy.

#### 3.3.9. High level aftereffects

Adaptation induced repulsive effects are known to happen at higher abstraction levels, e.g., face interpretation (Webster, 2011). Of course, such interpretations rely on higher level features than those considered here (tristimulus vectors, spatial, and motion contrast). Nevertheless, these higher level aftereffects have been interpreted in terms of shifting reference frames on these higher abstraction spaces (Leopold et al., 2001). An interesting avenue to explore would be gathering samples on those spaces and check whether the principles valid at lower abstraction levels are still valid.

#### 3.3.10. Computational complexity

One of the problems of the proposed technique is its computational cost and the number of samples required in high dimensional scenarios: see details in Section 4. The time consuming computation of local-to-global curves is a problem to obtain on-line versions of the algorithm. The cost could be alleviated by using recently proposed sequential approaches in which each nonlinear component is computed through regression (Laparra et al., 2014, 2015). Including density-based metrics, as those used in SPCA, would not substantially increase the complexity of such alternatives.

### 3.4. Final remarks

Our results represent new evidences that support the classical efficient coding explanation of visual illusions, as a result, we'd like to conclude with two appropriate classical quotes. In their seminal contribution, Weiss et al. (2002) concluded: …*we believe the underlying principle will continue to hold: that many [motion] illusions are not the result of sloppy computation by various components in the visual system, but rather a result of a coherent computational strategy that is optimal under reasonable assumptions*. And in their comprehensive review on [motion] perception, Burr and Thompson (2011) made this inspiring comment on that sentence: *No one has given better advice to anyone studying visual illusions*. Our normative model using SPCA to capture basic properties of visual signals is an additional confirmation of the above ideas (in our case focusing on the optimal encoding part of the problem) not only for motion, but also for texture and color.

One final *statistical* question: our brain may be misrepresenting reality just after sudden environment change, however, do these sudden changes really happen so often?

## 4. Materials and methods

Certain statistical approaches to explain aftereffects stress the need of multidimensional equalization to match the data manifolds in different adaptation conditions (Clifford et al., 2000; Clifford, 2002). The Sequential Principal Curves Analysis (SPCA) is a nonlinear unsupervised learning method designed to that general end (Laparra et al., 2012). The main advantage of SPCA nonlinear equalization with regard to linear techniques such as 2nd order decorrelation (PCA-based linear equalization), is that SPCA can be tuned for different statistical goals, and this can be used to check different organization hypothesis. Even though originally proposed in the context of color vision (Laparra et al., 2012), SPCA is general and, as shown in this work, it can be applied to data of other modalities. In this Section we explicitly check SPCA ability for different image texture data equalization: we show how it can be optimized either for information maximization or error minimization, leading to more sensible results than linear decorrelation. Below we outline how the response of SPCA sensors is computed, but all the technical details and code can be found at the Supplemental Material online (see Laparra and Malo, 2015 and http://isp.uv.es/after_effects).

### 4.1. Sequential principal curves analysis with tunable metric

Sensors (or features) identified by linear unsupervised learning are just straight lines in the signal space, e.g., the Principal Components in PCA (Jolliffe, 2002) or the Independent Components in linear ICA (Hyvarinen et al., 2009). The response to a stimulus of sensors identified in these linear ways is the projection of the stimulus onto the different vectors. The basic idea in SPCA is generalizing the set of linear sensors in PCA or ICA by using a sequence of Principal Curves (PCs) instead of a sequence of (straight) lines. Moreover, SPCA measures distances in these curves according to the local density of stimuli and different design strategies (infomax or error minimization). The response to a stimulus of SPCA sensors is the projection of the stimulus onto the curves.

As stated in Section 3.3, both strategies may be useful for biological systems to better transmit information (Laughlin, 1983), or to have undistorted representations of the outside world (MacLeod and von der Twer, 2001; Simoncelli, 2009). Moreover, both strategies differ from 2nd-order decorrelation. *Information maximization* is equivalent to looking for independent responses and a uniform use of the dynamic range of the sensors (Laughlin, 1983; Bell and Sejnowski, 1995). That is the general goal in nonlinear ICA (Malo and Gutiérrez, 2006; Hyvarinen et al., 2009). In contrast, *error minimization* tries to keep the representation error small in a scenario where the sensors have limited resolution or are subject to internal noise (MacLeod and von der Twer, 2001; Laparra et al., 2012). This is equivalent to the goal of transform coding or vector quantization with a restricted codebook size (Gersho and Gray, 1992).

**Figure 11:**
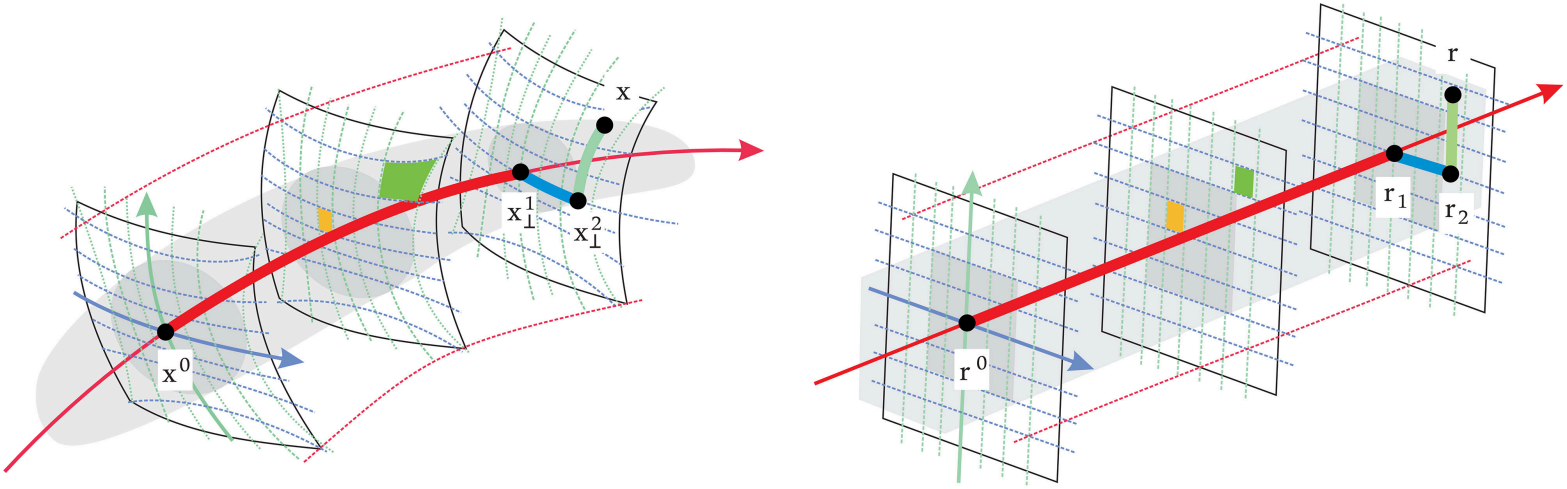
**The SPCA leading idea is removing redundancies by unfolding the dataset along the first and secondary Principal Curves (PCs) and performing local equalization along the way**. Here the first PC (Hastie and Stuetzle, 1989) is in bold red and the secondary PCs (Delicado, 2001) are in green and blue. Left plot represents the input domain x and right plot represents the response domain r. Gray regions represent the underlying PDFs. As in Self Organizing Maps (Kohonen, 1982), a curvilinear lattice is assumed (thin red, blue, and green curves). However, unlike in SOM, the computation of the whole lattice is not needed: to transform a certain x just the path in bold style, made of segments of PCs, is required. Moreover, the specific resolution per dimension emerges from data: the PDF-based metric in SPCA implies that highly populated regions in the input are expanded in the response while lower density regions are shrunk (e.g., orange and green areas). Given an origin, x^*o*^, in the first PC (red line) and some point of interest, x, the response for the point of interest is given by the lengths along this path: the first PC in red, the secondary PC in blue in the orthogonal subspace at ${\text{x}}_{⊥}^{1}$, which is the (geodesic) orthogonal projection of x on the first PC, and the secondary PC in green in the orthogonal subspace at ${\text{x}}_{⊥}^{2}$.

Figure 11 illustrates the SPCA concept, and Section 4.2 gives the expression to compute the responses (or projections): the identified sensors follow the curvature of the data and the discrimination metric depends on the probability density (Laparra et al., 2012; Laparra and Malo, 2015). The discrimination ability (or sensitivity) is high in highly populated regions and it is small in low density regions. This implies data equalization: high density regions in the input space, such as the area in orange, are expanded in the transformed domain, while the low density regions are shrunk in the response domain, e.g., the area in green. Note that the resulting multidimensional equalization may imply different resolution of a sensor (different SNR) depending on the activation of neighbor sensors, i.e., depending on the environment, which is the condition suggested in Stocker and Simoncelli (2006) to explain repulsive aftereffects.

### 4.2. Response of SPCA sensors

The SPCA transform (or response, *r*) is based on the integration of a signal-dependent Jacobian, ∇*R*(**x**), from a certain origin, **x**^*o*^, up to the stimulus, **x**, along a path made of segments of Principal Curves:

(4) $$r=R(x)=C\;\cdot \int_{x^{o}}^{x}\nabla R(x^{\prime })\;\cdot \;dx^{\prime }=C\;\cdot \int_{x^{o}}^{x}D(x^{\prime })\;\cdot \;\nabla U(x^{\prime })\;\cdot \;dx^{\prime }\;$$

where *C* is just a global scaling (constant diagonal matrix that independently scales each component of the response), and the Jacobian is made of (1) a local unfolding transform (a rotation *d***u** = ∇*U*(**x**) · *d***x** that looks for the local decorrelated directions), and (2) a local equalization, a diagonal scaling matrix *D*(**x**) [or *D*(**u**)] whose elements depend on the marginal PDF on the unfolded coordinates: $D{\left(u\right)}_{ii}\propto p_{u_{i}}{\left(u_{i}\right)}^{\gamma }$, where each marginal PDF is estimated from the samples in a *k*-neighbors ball.

The important features of SPCA are (i) the PDF-dependent discrimination metric tunable according to the exponent γ, and (ii) the path made of segments of PCs, which determine the curvilinear sensors. The first property allows using SPCA with Euclidean metric (γ = 0), with metric for optimal vector quantization or for sensors with limited resolution (γ = 1∕3) (Gersho and Gray, 1992; MacLeod and von der Twer, 2001), or with metric for maximum information transmission (γ = 1) (Gersho and Gray, 1992; MacLeod and von der Twer, 2001). The metric controls the relative relevance of highly populated regions. This ability to be tuned will be explicitly checked for visual texture patterns in Section 4.3.

Note that in the proposed path (Figure 11), the *i*-th PC is followed up to the geodesic projection of the point **x** on this PC, $x_{⊥}^{i}$. Here geodesic projections are understood as *projections that follow the local structure of the manifold*. The definition of locally orthogonal subspaces and geodesic projections are fundamental ingredients to ensure an accurate transform and inverse. The Supplemental Material available online (Laparra and Malo, 2015) describes the computation of these subspaces and projections and shows through experiments the accuracy of the transform. The selected path implies displacements in one PC at a time. Then, the vector *d***u**, which is tangent to the PC (Delicado, 2001), has a single non-zero component: the one corresponding to the considered PC at the considered segment. Therefore, the response of each sensor to the point **x** is just the length on each PC in the path, measured according to the metric related to the local PDF with the selected γ,

(5) $$r_{i}=C_{ii}\cdot \int_{x_{⊥}^{i-1}}^{x_{⊥}^{i}}D(x^{\prime })\;\cdot \;\nabla U(x^{\prime })\;\cdot \;dx^{\prime }=C_{ii}\int_{0}^{u_{i⊥}^{i}}p_{u_{i}}{\left({u^{\prime }}_{i}\right)}^{\gamma }\;d{u^{\prime }}_{i}$$

The scaling constants, *C*_*ii*_, are an arbitrary global response ranking or scaling of the dimensions in the response domain (e.g., to get a unit-volume hypercube in the right plot of Figure 11).

SPCA instrumentally requires an algorithm to draw a sequence of first (Hastie and Stuetzle, 1989) and secondary (Delicado, 2001) PCs *from specific points* (i.e., a local-to-global algorithm). Suitable choices include those in Delicado (2001) and Einbeck et al. (2005, 2010) or the one used here Laparra and Malo (2015). However, note that the choice to draw individual PCs is not the core of SPCA, but the PDF-related metric and the specific sequential path for the Jacobian integration leading to the curvilinear coordinate system (the nonlinear sensors).

Since SPCA is a generalization of PCA (or decorrelation), it is illustrative to see in which conditions SPCA reduces to PCA. This happens by (1) using Euclidean metric in the equalization along the PCs, e.g., using γ = 0, and (2) by increasing the rigidity of the principal curves so that they reduce to straight lines. Otherwise, SPCA sensors and responses will differ from those obtained through decorrelation.

### 4.3. Texture sensors using different metrics: nonlinear ICA vs. transform coding

Efficient representation of spatial information is a challenging problem for manifold learning techniques and a suitable scenario to check the effect of different optimality criteria. Here we present an example of applying SPCA to image texture data. The training set consists of 2 · 10^5^ four-dimensional vectors from 2 × 2 luminance patches of natural images from the calibrated McGill database (Olmos and Kingdom, 2004). SPCA was trained on these samples using infomax or error minimization metrics, i.e., γ = 1 or γ = 1∕3, respectively. The restricted 2nd-order decorrelation case, PCA, or rigid SPCA using γ = 0 is also considered for illustrative purposes. The learned representations were tested on the standard image Barbara (not included in the training set).

**Figure 12:**
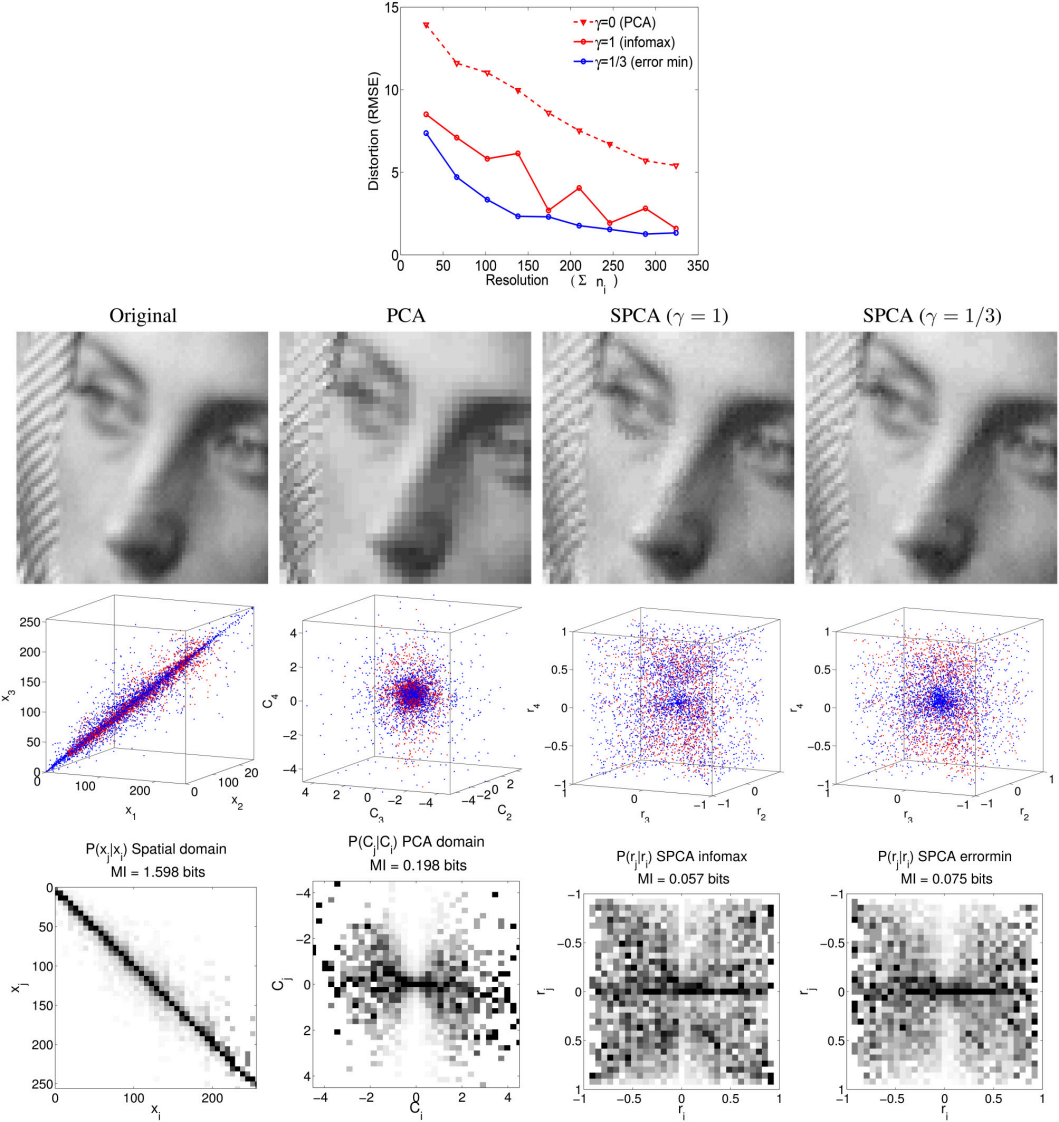
**Texture coding using conventional decorrelation (PCA) vs. infomax and error minimization (through SPCA)**. **Top** resolution-distortion plot. **Second row** reconstructed images using the same total number of bins, $\underset{i}{\sum }n_{i}=66$, in different representations. **Third row** training and test samples (in blue and red) in different domains. In the spatial domain the values represent luminance. In the PCA domain we showed the AC components and amplitudes were expressed in contrast as in Figures 4, 7. Four-dimensional histogram equalizations illustrated by the SCPA solutions are examples of the concept outlined in Figure 11. **Fourth row** conditional PDFs and mutual information (MI) independence measures in the considered domains. Dependence between coefficients implies visible structures in the conditional PDFs (bow-ties in the case of frequency coefficients). Note that greater MI values are consistent with visible (correlation or bow-tie) structures in the conditional PDFs.

The ability of SPCA for transform coding is illustrated by using sensors with limited resolution (uniform quantization in each dimension of the transformed domain) in the considered representations. In every case, resolution in each dimension was set according to standard bit allocation (Gersho and Gray, 1992). Figure 12 shows the reconstruction error as a function of the resolution of the sensors for 60 randomly chosen samples from the Barbara image. Figure 12 also shows reconstructed images from the quantized representations using the same sensor resolution (total number of quantization bins, or sum of bins per dimension). The resolution-distortion plot shows that SPCA with the *error minimization* metric substantially reduces the RMSE in image coding with regard to Euclidean metric (γ = 0, or uniform quantization of PCA) and to the *infomax* SPCA. The decoded images show the practical relevance of the numerical gain achieved by the non-Euclidean γ = 1∕3 approach.

Multidimensional equalization through SPCA is illustrated in the third row of Figure 12. It shows three (out of four) dimensions of the training and test samples in the spatial domain, in the PCA domain, and in the SPCA domains with γ = 1 and γ = 1∕3. While *infomax* SPCA leads to an approximately uniform PDF by expanding the central portion of the PCA domain and contracting the high-contrast region, the center of the domain is not expanded that much in *error minimization* SPCA: in the latter, the saturating nonlinearities are less sharp, consistently with the responses shown in Figure 8.

The ability of SPCA for nonlinear ICA is qualitatively and quantitatively assessed by inspecting the conditional PDFs between nonzero frequency coefficients, as in Buccigrossi and Simoncelli (1999), Hyvärinen et al. (2003), and Malo and Laparra (2010), and by the corresponding mutual information (MI) measures. Bow-tie structures in the conditional PDFs and MI measures in the spatial domain and in the PCA domain are consistent with previously reported results for natural images (Malo and Laparra, 2010). Uniform conditional PDF and small MI show that SPCA with the *infomax* metric strongly reduces the redundancy between the coefficients of the representation. In contrast, in the case of SPCA with the *error minimization* metric the bow-tie shape is still visible in the conditional PDF. Equalization implies maximum independence between components (lower MI), but in order to minimize the error under quantization, high contrast outliers have to get relatively more resolution than in simple equalization (as is the case with γ = 1∕3).

These results confirm the SPCA theory in the case of visual textures: the multidimensional equalization can be tuned to optimize different perceptually meaningful criteria, either *infomax* (using γ = 1) or *error minimization* (using γ = 1∕3). Moreover, note that the uniform quantization transform coding example (that leads to amplitude dependent noise in the PCA domain) also has biological meaning. On the one hand, amplitude-dependent noise in frequency representations of texture makes sense: psychophysical models usually assume uniform resolution after the nonlinear mechanisms (Watson and Solomon, 1997; Hillis and Brainard, 2005; Laparra et al., 2010), and this is equivalent to Poisson-like noise in linear neurons (Georgeson and Meese, 2006). On the other hand, when assessing the perceptual plausibility of different models, the more plausible is the one that is able to *hide* more noise injected in the internal representation (Freeman and Simoncelli, 2011). Generation of metameric textures by adding noise in the representation domain, as illustrated in the transform quantization example, may be used to further analyze the validity of the proposed models.

## Author contributions

VL contributed to the development of SPCA and to the analysis of the experiments. JM conceived the work, prepared the data and code for the experiments, and contributed to the interpretation of the results.

### Conflict of interest statement

The authors declare that the research was conducted in the absence of any commercial or financial relationships that could be construed as a potential conflict of interest.
